# Factors influencing access, quality and utilisation of primary healthcare for patients living with hypertension in West Africa: a scoping review

**DOI:** 10.1136/bmjopen-2024-088718

**Published:** 2024-12-20

**Authors:** Kezia Naa Amerley Akosua Amarteyfio, Eugene Paa Kofi Bondzie, Veronika Reichenberger, Nana Efua Enyimayew Afun, Albert Kofi Mensah Cofie, Mary Pomaa Agyekum, Paul Lamptey, Evelyn K Ansah, Irene Akua Agyepong, Tolib Mirzoev, Pablo Perel

**Affiliations:** 1 Ghana College of Physicians and Surgeons, Accra, Ghana; 2 Public Health, Ghana College of Physicians and Surgeons, Accra, Ghana; 3 Centre of Global Change and Health, London School of Hygiene and Tropical Medicine, London, UK; 4 Ashesi University College, Berekuso, Greater Accra, Ghana; 5 Dodowa Health Research Centre, Accra, Ghana; 6 Ashesi University, Berekuso, Greater Accra, Ghana; 7 Research and Development Division, Ghana Health Service, Accra, Ghana; 8 Public Health Faculty, Ghana College of Physicians and Surgeons, Accra, Greater Accra, Ghana; 9 Dodowa Health Research Center, Ghana Health Service Research and Development Division, Dodowa, Greater Accra, Ghana; 10 Global Health and Development, London School of Hygiene and Tropical Medicine, London, UK; 11 EPH, LSHTM, London, UK

**Keywords:** Hypertension, Primary Care, Health Services Accessibility, Patient-Centred Care, QUALITATIVE RESEARCH

## Abstract

**Abstract:**

**Objectives:**

Hypertension is one of the most prevalent non-communicable diseases in West Africa, which responds to effective primary care. This scoping review explored factors influencing primary care access, utilisation and quality for patients with hypertension in West Africa.

**Design:**

Scoping review using the Preferred Reporting Items for Systematic Reviews and Meta-Analysis extension for scoping reviews.

**Data sources:**

Published literature from PubMed, Embase, Scopus, Cairn Info and Google Scholar, between 1 January 2000 and 31 December 2023.

**Eligibility criteria:**

Systematic reviews, observational studies and reports involving participants aged 18 years and above, written in English, French or Portuguese, were included. Clinical case series/case reports, short communications, books, grey literature, randomised control trials, clinical trials, quasi-experiments, conference proceedings and papers on gestational hypertension and pre-eclampsia were excluded.

**Data extraction and synthesis:**

Data from included studies were extracted onto an Excel spreadsheet and synthesised qualitatively using thematic analysis structured by the components of the overall review question.

**Results:**

The search yielded a total of 5846 studies, 45 papers were selected for full review and 16 papers were eventually included. Macro (contextual) barriers included economic, funding and geographical barriers. Meso (health system) factors include access to medications, tools, equipment and other supplies, out-of-pocket payments, availability of health insurance, health workers numbers, capacity and distribution. Micro (community and patient factors) barriers included financial barriers and limited knowledge, whereas facilitators included the availability of alternative providers and community and household support. These factors are interconnected and complex and should be addressed as a whole to reduce the burden of hypertension in West Africa.

**Conclusion:**

Multiple complex and interrelated factors at contextual, health systems, community and patient levels act as barriers and enablers to access, utilisation and quality of primary care for hypertension in West Africa. Improving primary care and outcomes will, therefore, require multilevel multifaceted interventions.

**STRENGTHS AND LIMITATIONS OF THIS STUDY**

- The scoping review employed rigorous steps in searching for data, selection, screening and inclusion of studies.
- The search strategy included articles written in the three official languages used in West Africa (English, French and Portuguese) allowing for diversity of population studies and possible generalisability.
- Only published literature was reviewed, leaving a possibility of some omissions from the grey literature sources.
- No quality assessment of the studies included was conducted, which could present a challenge to assessing the rigour of the reviewed evidence base and could be addressed in further research.

## Introduction

 Cardiovascular disease is one of the leading causes of morbidity and mortality in sub-Saharan Africa (SSA).^1^,^3^ Hypertension is considered a leading risk factor globally and in SSA.^4 5^ Globally, an estimated 1.28 billion adults between the ages of 30 and 79 are living with hypertension, and about two-thirds of this number live in low- and middle-income countries (LMICs).^6^ In a study involving over 110 414 patients in SSA, there was a pooled prevalence of 30% for hypertension between the ages of 31 and 76; and only 7% had controlled blood pressure.^7^ Hypertension prevalence globally is projected to increase to 44% in 2030.^8^ An estimated 74.7 million individuals in SSA are hypertensive and this number is projected to increase to 125.5 million by 2025.^9^ Hypertension is prevalent in the West African subregion as well^10^,^12^ comprising 15 LMICs (Benin, Burkina Faso, Cabo Verde, Cote d'Ivoire, The Gambia, Ghana, Guinea, Guinea-Bissau, Liberia, Mali, Niger, Nigeria, Senegal, Sierra Leone and Togo).^13^

Hypertension is a condition that can be well managed with effective access to adequate quality of primary healthcare (PHC).^14^,^16^ By PHC for hypertension, we refer to the prereferral often ambulatory level of health promotion, prevention and first-level personal care available at the community, subdistrict and district levels. It is the first point of contact with the health system for most patients.^17^ Health facilities at this level typically include health centres, small clinics and polyclinics. Access and utilisation of quality PHC for hypertension in West Africa are often low for multiple reasons, such as cost, lack of knowledge, essential equipment, staff and supplies and medicines.^18 19^ Individual patient factors, such as medication non-adherence, use of traditional medicines, cultural beliefs and lack of knowledge, also affect how well their blood pressure is controlled.^20^,^22^

A recent scoping review explored and synthesised the reasons for poor blood pressure control in Eastern SSA, focusing on patient, professional, PHC system and public health policy challenges.^23^ However, there is no similar study for western SSA. Also, the study in Eastern SSA did not explore contextual factors that influence access, utilisation and quality of primary care for hypertension. This study addresses these knowledge gaps by exploring and synthesising the available literature on the barriers and enablers to PHC for the control of hypertension in West Africa. We hope that the findings of this study will be useful to policymakers in identifying key health system interventions to improve the control of hypertension in West Africa.

### Review aims and objectives

This review aimed to identify and analyse the factors that hinder or enable the access, utilisation and quality of PHC for patients living with hypertension in West Africa and how and why they work, to contribute evidence for strengthening hypertension control at the PHC level.

The specific objectives of this review were as follows.

1. Identify the factors documented in the literature that hinder or enable the management of hypertension at the PHC level in West Africa (including access, utilisation and quality of care) and analyse how and why they work, covering: (1) contextual factors, such as national and global, sociocultural, socioeconomic and political; (2) health facility and health system factors and (3) individual/patient factors.
2. To identify and highlight outstanding gaps for further research on the PHC of hypertension in West Africa.

### Conceptual framework

**Figure 1:**
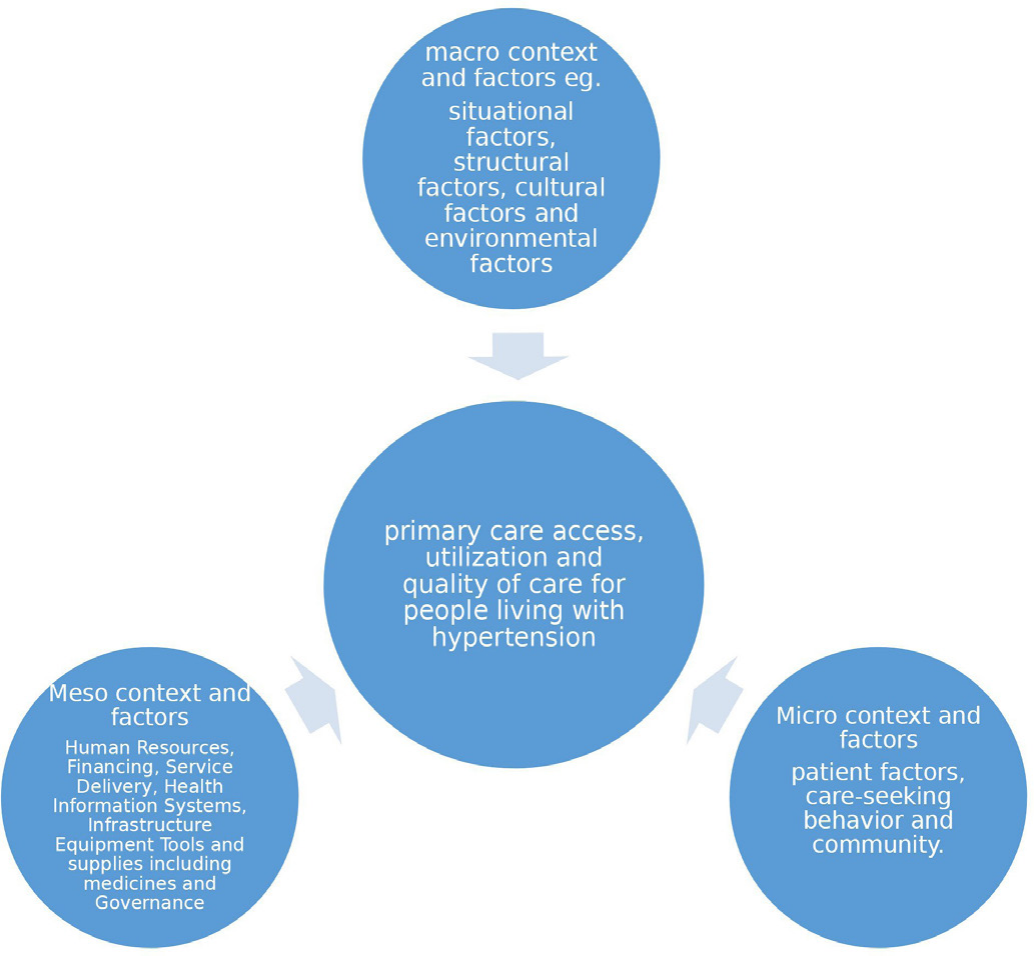
Framework on the factors that influence primary care access, utilisation and quality for people living with hypertension.

A conceptual framework of factors that influence PHC for people living with hypertension in West Africa was used to help structure our review. This framework (figure 1) was developed based on Leichter’s framework for analysing context, the WHO building blocks framework for health systems^24^ and also drew on insights from empirical studies on the subject,^23 25 26^ including a study on facilitators and barriers to non-communicable disease (NCD) prevention in Pakistan.^27^ The four categories of context from Leichter’s framework^26^ include: (1) situational factors, such as wars, famine, epidemics and political instability; (2) structural factors, such as economic structure, political system, technological change, degree of urbanisation, the structure of the labour markets and demographic structure; (3) cultural factors, such as the level of literacy, and values on issues, such as religion, gender inclusion/participation and corruption and (4) environmental or international/exogenous factors external to the political system, such as the role of transnational companies and international agreements and events. This framework was used due to its extensive assessment of contextual factors that influence health. The WHO’s six building blocks comprise service delivery, health workforce, health information systems, financing, essential medicines and supplies, and leadership and governance.

## Methods and analysis

The detailed published protocol for this scoping review is available elsewhere^28^ and accessible at the following address: http://doi.org/10.1136/bmjopen-2023-077459

### Inclusion criteria

Relevant papers written between 1 January 2000 and 31 December 2023 with participants 18 years and above were included. Articles, systematic reviews, observational studies and reports that included information on the relevant factors that influence primary care management of hypertension in West Africa were included in the paper. Papers written in English, French and Portuguese were included.

### Exclusion criteria

Clinical case series/case reports, short communications, books, grey literature, systematic reviews, randomised control trials, clinical trials, quasi-experiments and conference proceedings were excluded. Papers on gestational hypertension and pre-eclampsia were also excluded.

### Patient and public involvement

No patients were involved in the development of the review.

### Identifying relevant studies

Searches were conducted using PubMed, Embase, Scopus, Cairn Info and Google Scholar databases for the period of 1 January 2000–31 December 2023 by authors KNAAA, EPKB, NEEA, MPA and VR. The Google Scholar was included alongside more traditional databases to ensure capturing papers from newer journals, which may not yet have registration in academic databases. This time frame was selected because there has been a surge in the burden of NCDs in SSA over the past two decades, driven by increasing incidence of cardiovascular risk factors, such as unhealthy diets, reduced physical activity, hypertension, obesity and diabetes.^1^ The key concepts from our research question and alternative search terms (see online supplemental appendix 1) were combined using the Boolean terms AND, NOT and OR. Excel power query was used to generate all combinations of the key search terms to perform an exhaustive search. The search strategies used for the various databases are shown in Appendix 2. The search strategy was adapted as appropriate for each of the search databases. Only published literature was included in the review. The search results were exported into Rayyan software for the removal of duplicates and abstract screening.

### Study selection

**Figure 2:**
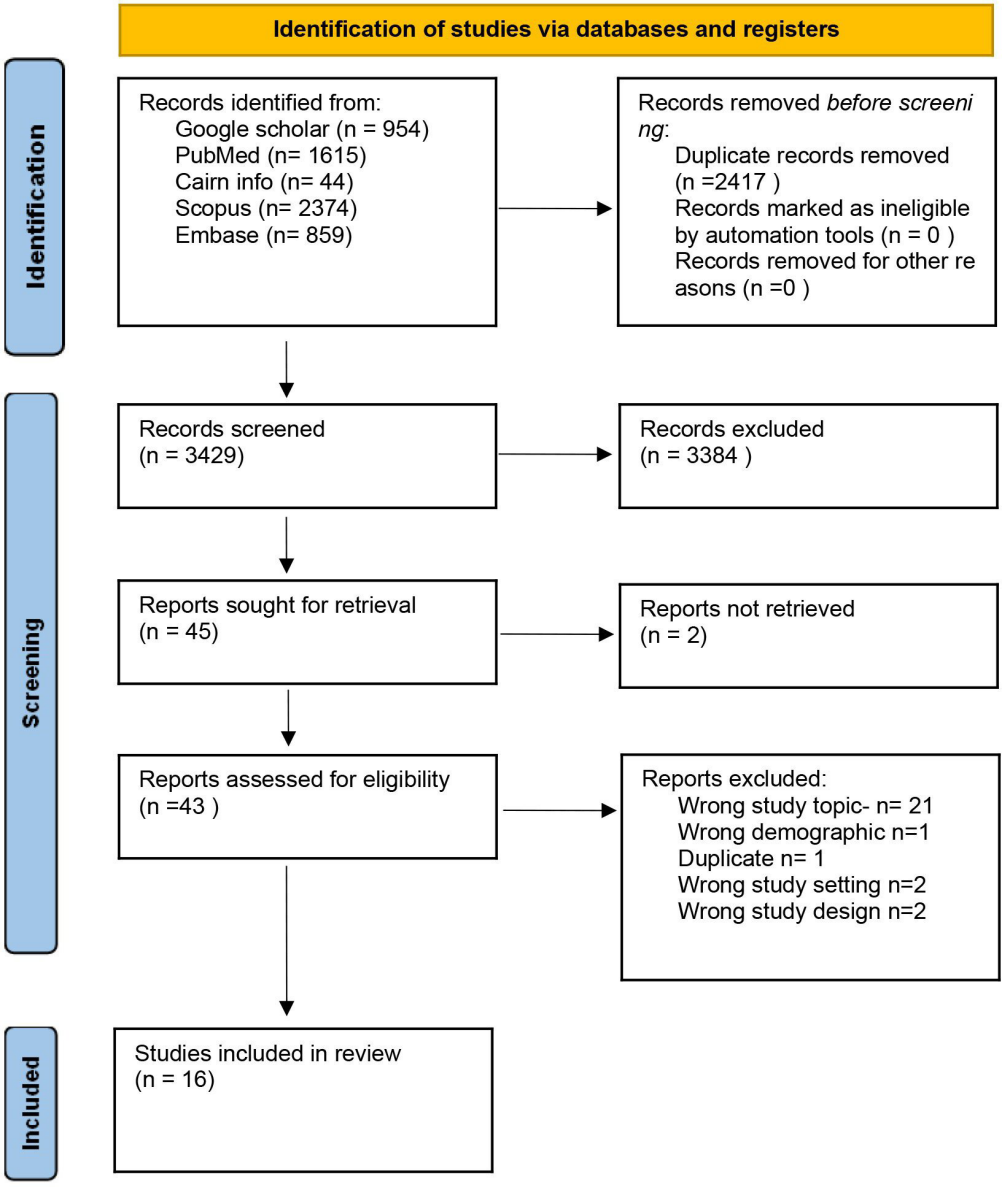
PRISMA 2020 flow diagram for scoping review. PRISMA, Preferred Reporting Items for Systematic Reviews and Meta-Analysis.

The studies found were screened against the eligibility criteria and study selection was done by two independent researcher pairs. Screening was done by authors KNAAA and EPKB, NEEA and VR, and PL and AKMC. Screening followed two stages, first, using the study titles and abstracts, and subsequently based on reading of full texts. Any disagreements were resolved through discussion and, where necessary, a third independent researcher was involved. There was an initial pilot selection of a few papers to test the search eligibility criteria prior to the start of the main selection. This was done by two independent reviewers to ensure reliability. The results of the selection are presented in a Preferred Reporting Items for Systematic Reviews and Meta-Analysis (PRISMA) flow diagram,^29^ as shown in figure 2. A PCCS framework, which stands for Population, Concept, Context and Study elements, is outlined below^30^ and guides the screening and identification of relevant studies. This framework was used rather than a population, intervention and comparison outcome framework because the research question was less clinically focused with no measure of a direct intervention or outcome.

Population: adults 18 years or older with systemic hypertension in West Africa.

Concept: hindering and enabling factors that influence primary care for hypertension.

Context: macro (National- and global-level factors), meso (health system factors) and micro (patient and community factors).

Study: qualitative, quantitative, meta-analysis, social science case studies and case series.

### Data extraction

The data were entered into a ‘data charting form’ using the database programme Excel. The data charting form included the title and author(s), year of publication, period the data was collected, type of paper, the aim of the study, study design, methodology, location where study data was collected, sample size, study objectives, key findings (macro, meso and micro level), comments, limitations, recommendations and others. Two independent reviewer pairs tested the data charting form on a few studies to ensure validity. All disagreements were settled by consensus or through the involvement of a third independent reviewer.

### Data synthesis

The data were extracted and, subsequently, synthesised for analysis and categorised as context (macro), health systems (meso) and patient and community (micro) factors. Contextual factors were categorised as per our conceptual framework into situational, structural, cultural and environmental/exogenous factors. Health system factors were categorised by the six WHO health systems building blocks of governance (human resources, financing, service delivery, health information systems, infrastructure equipment tools and supplies, including medicines). Thematic narrative analysis approach was used and the major themes arising from each group were presented in the results.

## Results

The search yielded a total of 5846 studies. After the removal of duplicates, 3429 papers remained. Using a study selection flowchart (Appendix 3), we screened the papers based on their titles and abstracts. 45 papers were extracted for full paper review after titles and abstract screening. 16 papers were finally included. The reasons for the exclusion of 27 papers are summarised in the PRISMA chart (figure 2). Two papers could not be obtained via open access or institutional subscriptions.

Of the 16 included studies, 10 were from Nigeria, 3 from Ghana, 1 from Benin, 1 from Sierra Leone and 1 paper was conducted in both Ghana and Nigeria. The study by Adedapo *et al*^31^ was included despite an incomplete paper as the available copy contained the study results and a part of the discussion. A summary of the key findings from the included studies is in the online supplemental table 1.

### Contextual (macro level) factors

Different situational, structural, cultural and environmental factors from Leichter’s framework^26^ emerged from our analysis. Five studies highlighted the structural factors (economic structure, political system and demographic structure)^1931^,^34^ and two studies highlighted economic and financial facilitators and barriers to NCD care access, utilisation and quality.^31 32^

Studies showed that *economic structures*—specifically the inadequate prioritisation of and allocation of resources to NCDs in government funding—hinder access to and utilisation of PHC for hypertension.^31 32^ In a study in Ghana, policymakers indicated that *current health funding priorities* were maternal and newborn mortality, sanitation, malaria and other infectious diseases.^32^ Although NCDs were recognised as a major public health challenge, they were not given priority in allocating scarce resources.^32^

Several studies highlighted the importance of *access to medicines*. In a study from Nigeria, a preceding (2009–2010) policy, where all medications were given for free, was replaced with an annual limit for each staff or family member, beyond which a copayment had to be made. An incentive of up to 50% of the medical allowance was returned to any staff that did not fully use their allowance during the period. Although there was observed to be an increase in the number of patients achieving full blood pressure control, utilisation declined.^31^ The interpretation of the observations was, however, not conclusive, since though the policy change could have created a financial barrier to accessing healthcare, there was also a drop in the catchment population, and it was not clear if it was the policy change or the drop in catchment area population explained the drop in utilisation after the policy change.

In Ghana, hypertension is included in the minimum healthcare benefits provided by the National Health Insurance Scheme (NHIS). To access its benefit, citizens have to pay an annual premium, with children under 18 and the elderly being exempt. Constraints to the payment of enrolment and subsidised annual premiums were observed.^32^

Another policy-related barrier mentioned by two studies was the limitation on non-physician health workers in prescribing medications. Given the limited number of physicians, this reduced access to primary care and medications.^32 33^

*Geographical barriers to accessing hypertension care* included factors related to numbers, types and location of health facilities, as well as road and transportation access. Seasonal changes were found to hinder access to and utilisation of PHC for hypertension. Healthcare facilities are more likely to be accessed and used by clients when they are nearby; therefore, long distances to facilities and poor road networks create significant barriers to accessing hypertension care.^19 34^ In Ebonyi, Nigeria, about two-thirds of the respondents said that the healthcare facility was accessible because it was within an hour’s walk from their homes. However, only about a third of them mentioned that it was easy for them to get to the facility where they receive treatment for hypertension.

Our analysis revealed that no studies explicitly reported findings with regards to situational, cultural and international factors in hypertension care at the PHC level.

### Health system (meso level) factors

*Human resource numbers and distribution* were important factors influencing access, utilisation and quality of PHC. Insufficient number of health workers was mentioned in five studies.^3233 35^,^37^ Apart from numbers, challenges with inequities in distribution were mentioned.^32^ Areas with closer proximity to training schools for health workers were observed to have a higher availability of health workers in the primary health facilities.^35 38^ In a study in Ghana, an increase in doctor density led to an improvement in the quality of care received by hypertensive patients evidenced by a reduction in hospitalisation rates for hypertension patients.^37^

Adequate *provider knowledge of and adherence to guidelines as well as the availability of decision-making support systems* enables better hypertension management.^39^ Conversely, lack of training and awareness of treatment guidelines by health workers was a barrier to the quality of primary care mentioned in multiple settings.^3335 36 38^,^40^ In one study, despite all the non-physician healthcare providers at the study site having training on how to measure blood pressure, only about 40% of them were aware of the correct treatment goal for uncomplicated hypertension.^41^ Another study also reported that care protocols and education materials for the prevention of cardiovascular diseases were scarcely available in health centres.^40^

Our review revealed multiple *medical equipment and supplies-related* facilitators and barriers to hypertension care in West Africa. The limited availability of equipment, tools and supplies, including medicines, was a barrier to care for hypertension.^42^ Essential medications were found to be unavailable in PHC facilities in five studies.^19 32 33 35 41^ In one study from Ghana, essential antihypertensive medication like Lisinopril, Atenolol, Carvedilol and spironolactone were unavailable and primary care facilities had to procure these from external sources like private pharmacies.^43^ In five studies, tools required for hypertension screening and monitoring were not readily available in PHC facilities, in the needed quantities.^19 33 39 41 42^ The lack of equipment led to opportunistic screening rather than screening for all members of the community.^32^ In one study, some health workers had to use personal equipment to provide services at their primary health centres because the government did not provide them.^35^

*Health financing arrangements* related to how clients paid for services influence access to and utilisation of PHC facilities for hypertension. Individuals from lower socioeconomic backgrounds were more affected by financial barriers to care^33^; with out-of-pocket payments at the point of service being particularly challenging for many.^43^ The availability of health insurance helps to reduce the barrier posed by out-of-pocket payments at the point of service use.^32 36^ In a study in Ghana and Nigeria, participants with health insurance coverage were 2.6 times more likely to have controlled blood pressure and had 4.5 times better medication adherence than patients without insurance.^44^ However, health insurance coverage was low and challenges of delayed reimbursements affected its effectiveness.^32 36 44^

In the area of *service delivery*, although hypertension service availability and readiness assessment (SARA) surveys in Nigeria met the general service criteria, the services were instituted to manage infectious diseases and not NCDs.^38^ The SARA studies in Nigeria found there was availability of blood pressure measuring equipment in most PHC facilities, which allowed for screening and diagnosis; however, most respondents from other studies in Nigeria noted equipment shortage as a barrier to hypertension care.^33 35 38 40^

The different health systems factors were interrelated and mutually reinforcing. Poor infrastructure, inadequate supervision, ineffective referral processes and varying/inappropriate interpretations of national guidelines all affected the consistency and quality of the delivery of hypertension and diabetes care mentioned by community health workers in studies in Nigeria.^33 36^

None of the reviewed papers explored health information systems and leadership and governance as potential barriers or facilitators to PHC management of hypertension.

### Micro (patient, household and community level) factors

*Patient knowledge, education and empowerment* emerged as important aspects of how individuals make decisions about their health. Lack of knowledge about hypertension treatment, appropriate diets, risk factors and drug adherence served as barriers to patients’ access and utilisation of PHC for hypertension.^32 41^

Out-of-pocket fees as an access and utilisation barrier were mentioned in multiple studies.^19 32 40 43^ As set out earlier, these barriers are worse for people from lower socioeconomic backgrounds with no health insurance. They also constrained adherence with some patients resorting to buying their medicines only as and when they can afford them.^34^ In one study in Nigeria, all 145 respondents could not afford their antihypertensive medications. The median monthly income was 8000 Nigerian Naira, whereas the median monthly cost of antihypertensive medications was 3500 Nigerian Naira.^19^

The availability of multiple *alternative formal and informal care providers* influenced access, quality and utilisation. In one study, patients generally used primary care facilities, making it a good avenue for interventions to improve hypertension care.^41^ In another study, the perceived curative effects of herbal medicines, compared with the requirements for lifelong adherence to allopathic treatments, encouraged a preference for the use of herbal medicines.^32^ Factors that influenced medicine adherence included the use of alternative medicines, non-attendance to reviews, forgetfulness and poor knowledge about hypertension management.^34^

Our review also highlighted the importance of *social support* in determining access to hypertension care. In one study, community and household support was an important enabler for medication adherence and attending follow-up appointments, eventually leading to patients having better-controlled blood pressure.^45^

Our analysis also revealed different knowledge gaps that can be usefully addressed in future research to inform improved hypertension control in West Africa. As mentioned earlier, limited published knowledge exists concerning different factors at the macro (situational, cultural and international factors) and meso (information systems and leadership arrangements) levels. Most studies found were from Ghana and Nigeria, which limits the generalisability of the review findings. There was generally limited research on hypertension policy implementation at the PHC level.

## Discussion

This review sought to explore the factors that hinder or enable access, utilisation and quality of primary care for hypertension management in West Africa. We have identified multiple factors at contextual or macro, health systems or meso and community and individual or micro levels. At the contextual level, they include economic and funding structures and priorities, as well as factors related to other systems, such as roads and transportation. At the health systems level, they include human resources numbers, skills and distribution, access to medication, equipment and supplies and geographical barriers related to the location and distribution of facilities. The type of health financing arrangements such as the extent of out-of-pocket payments at point-of-service use versus social insurance or similar systems is also an important health systems barrier. Key patient- and community-level factors include knowledge, education, ability to pay, availability of alternative care providers and the existence of social support networks.

These factors are interrelated and act as barriers or enablers to access, utilisation and quality of primary care depending on how they are structured and organised in context, and were similar across all the countries studied that is, Ghana, Nigeria, Benin and Sierra Leone. Thus, for example, although Ghana has an NHIS with over half the population covered and better protection against out-of-pocket fees for the insured, delayed reimbursements led to implementation challenges that effectively exposed some clients to out-of-pocket fees. That some of the population remained uncovered also limited full effectiveness even when an insurance scheme was in place, as for example, in the paper from Nigeria where insurance coverage in 2016 was reported as less than 5% due to difficulty in convincing autonomous state governments to buy into the scheme, inadequate health workforce, poverty and low level of awareness at the community level among others.^46 47^ Low health insurance coverage is common across SSA. Only Gabon, Ghana and Rwanda have extended national coverage to a significant portion of their populations (40.8%, 57.7% and 78.7%, respectively).^48^ The literature highlights that tackling contextual, health system, community and individual factors together is likely to be a better approach to effective interventions to improve PHC for hypertension.^46 47^

Concerning the availability of blood pressure measuring equipment in most PHC facilities in Nigeria, it was interesting to note the contrast in responses between the healthcare workers and the SARA surveys. The contrast may be due to the equipment being available but not functional at the primary care facilities. There may also be inconsistencies in the reporting of service availability on the ground.

The knowledge gap among healthcare workers has a great impact on the quality of patient care. Measuring the blood pressure of patients without proper interpretation of the results can lead to gaps in patient care with many patients over or underdiagnosed. Provision of accessible educational materials like clinical algorithms and straightforward flowcharts at the healthcare centres can help improve the knowledge of healthcare workers. Regular training sessions and refresher courses for healthcare workers can also ensure accurate and updated practices. Exploring task-shifting interventions as used in the community-based hypertension improvement programme in Ghana can help with the issue of insufficient health workers in primary care facilities.^32 49^ Task shifting, or redistributing care from physicians to nurses and lower level workers, has shown positive results in improving access to healthcare for NCDs.^50 51^

Patient- and community-related barriers and facilitators are noteworthy, especially for people-centred health systems. There is substantial literature on community engagement and involvement in healthcare, including effective interventions and measures to address limited knowledge through targeted educational programmes and ensure the empowerment of patients and their carers with regards to decisions related to NCD control.^52 53^ While the existence of social support networks is an important facilitator of hypertension care, which should be leveraged in ensuring continuous support to people living with hypertension, the availability of alternative care providers highlights a possible call for integrating traditional and allopathic healthcare, which is particularly important in cultural, religious and traditional West African societies.

**Figure 3:**
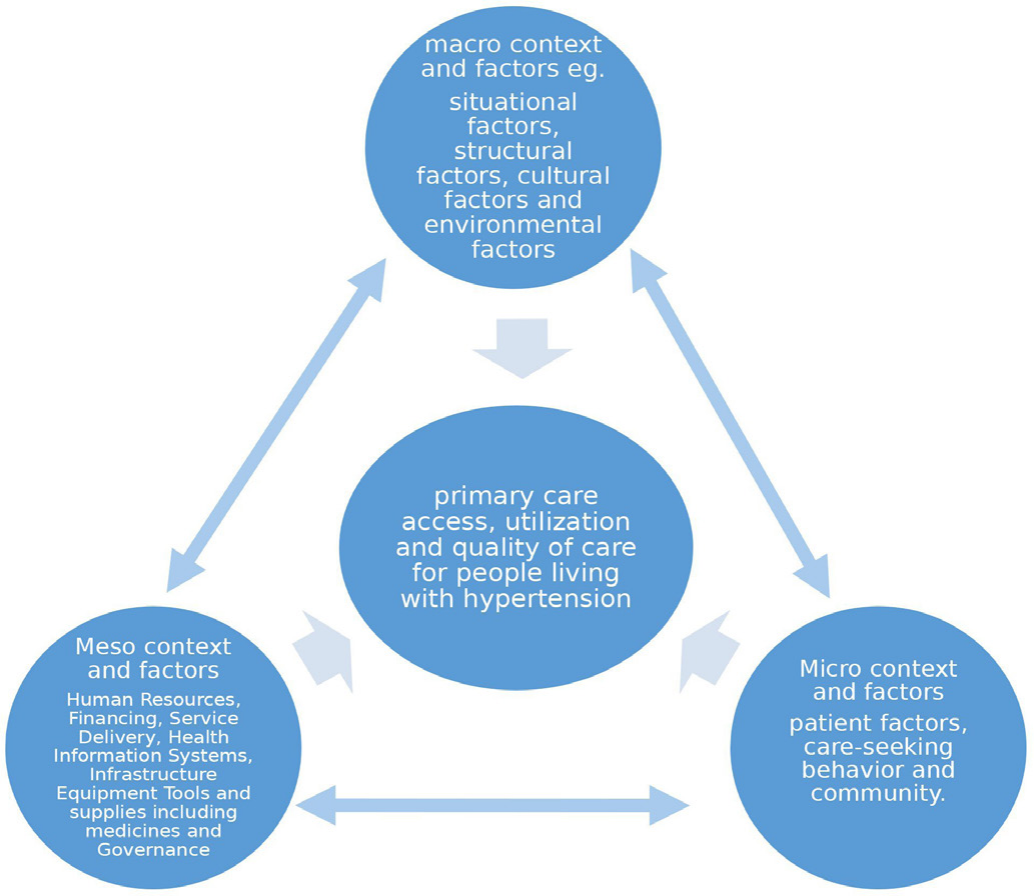
The framework on the factors that influence primary care access, utilisation and quality for people living with hypertension shows interconnectedness.

The findings of our study generally validate our conceptual framework but also highlight possible modifications, as shown in figure 3. For example, our original framework had insufficient consideration of the explicit interconnectedness of the factors such as health worker density due to proximity to training schools and multiple health systems influences on the quality of hypertension care at the PHC level reported earlier. Despite the presence of an NHIS, poor implementation at the health system level led to patients having to pay out-of-pocket for medications and medication unavailability, thus highlighting the complexity of improving medication access.^2325^,^27^

This study has the following strengths and limitations. The review has helped to identify information available and gaps in the published literature on factors affecting access, quality and utilisation of primary care for control and management of hypertension in West Africa that can inform the production of interventions. This review highlights the relatively limited published literature with available publications dominated by Anglophone West Africa. A potential limitation of this study is that most of the studies included were done in Nigeria (10/16) followed by Ghana, which has a similar health system and socioeconomic structure. There may be a challenge in the generalisability of the findings to all of West Africa. One of the closed-access papers that could not be included in this review was a study in Nigeria, which would have shed more light on the patient and caretakers’ experiences with hypertension. The other study was done in Senegal, which is an under-represented context in the current literature. It would have been useful to explore the barriers there. However, it is likely that the findings would have been similar and complemented those from the included studies. An area with potential for future research is on situational, cultural and international factors that influence hypertension care at the PHC level.

A key implication for improved policy and practice of hypertension control is the need to explore multifaceted rather than unidimensional interventions, addressing multiple barriers across the macro–meso–micro levels. Unidimensional interventions such as improving health workers' capacity (knowledge and skills), prioritising NCD in government funding for service delivery at PHC, replacing out-of-pocket payments at point-of-service use with some form of insurance and improving availability of equipment, tools and supplies can all make a difference. However, given the inter-relationships and synergies illustrated in our conceptual framework, synergistically combining interventions into an integrated system of care is likely to produce an effect that is greater than the mere sum of putting in place individual non-synergised interventions.

### Conclusion

Multiple complex and interrelated factors at contextual, health systems, community and patient levels act as barriers or enablers to access, utilisation and quality of PHC for hypertension in West Africa, and the details of how an intervention is implemented rather than just the intervention idea matter. Improving PHC and outcomes will require well-integrated multilevel multifaceted interventions.

## supplementary material

10.1136/bmjopen-2024-088718 online supplemental file 1

10.1136/bmjopen-2024-088718 online supplemental file 2

## Data Availability

All data relevant to the study are included in the article or uploaded as supplementary information.
