# Supplementary material for: Factors influencing access, quality and utilisation of primary healthcare for patients living with hypertension in West Africa: a scoping review
Source: BMJ Open. 2024 Dec 20;14(12):e088718. doi: 10.1136/bmjopen-2024-088718 (PMC11667391; doi:10.1136/bmjopen-2024-088718)
Supplement: online supplemental file 1 [file bmjopen-14-12-s001.pdf]

Table 1-Summary of included studies

Papers title, first author and year of publication	Sample size	Study participants	Aims and objectives	Key findings
1. Anti-hypertensive medication access and affordability and their association with blood pressure control at a teaching hospital in Ghana.<br>Harrison MA et al 2021	310	hypertensive patients	To determine the affordability and accessibility of antihypertensive medicines and their association with blood pressure control in hypertensive patients.	**Meso factors-** 60% of the anti-hypertensive medicines were available at the polyclinic pharmacy. Lisinopril, Atenolol, Carvedilol, and spironolactone were not available.<br>- A significant proportion of participants faced interruptions in accessing their anti-hypertensive medication.<br>- Health Insurance Influence: Patients obtaining medicines via health insurance were more likely to achieve blood pressure control than those making out-of-pocket payments or using a combination of health insurance and out-of-pocket payments.<br>**Micro factors-** About 13% (n=39) of patients had zero capacity to make any out-of-pocket payments for medicines. Less than 20% of patients could pay for antihypertensives < USD 21. Those who could afford their medication were more likely to have controlled blood pressure.<br>- The association between hospital access and blood pressure control suggests that accessing medication from the hospital positively influences the likelihood of achieving controlled blood pressure.
2. Awareness of hypertension guidelines and the diagnosis and evaluation of hypertension by primary care physicians in Nigeria.<br>Ale O, 2017	403	Lagos bases PCPs (general practitioners)	1. To evaluate awareness of hypertension among Primary care practitioners in Lagos, Nigeria, and its effect on their diagnostic approach to hypertension. 2. To determine the relationship between the type of practice, namely private or government, and hypertension diagnosis and work-up.	**- Meso factors -** Less than half of the respondents in this study (46.7%) were aware of the hypertension guidelines.<br>- Only 26.4% of all respondents allowed a rest of 10 minutes or more, as recommended by the IFHA guidelines<br>- As expected, the PCPs in the guideline-aware group performed better than those in the unaware group (seven out of 19 responses vs two out of 19 responses). This shows that hypertension guideline awareness is associated with better hypertension care.
3. Barriers to management of	1000	Hypertensives	To assess the capacity of	**Meso factors-** Inexpensive generic antihypertensive drugs are not

cardiovascular risk in a low-resource setting using hypertension as an entry point.<br>Shanthi Mendis, 2004	hypertensives attending the selected facilities for follow-up, and healthcare providers; 43 healthcare facilities	attending primary and secondary level health care and private health care facilities.	healthcare facilities in a low resource setting to implement the absolute risk approach for assessment of cardiovascular risk in hypertensive patients and effective management of hypertension	available in most facilities.<br>- Stethoscopes and weighing scales were available in 93 and 70% of facilities. Mercury, aneroid, or digital sphygmomanometers were available in 74, 30 and 7% of facilities, respectively.<br>- A regular supply of anti- hypertensive drugs was available only in 49% (21/43) of facilities.<br>- Investigations to exclude secondary hypertension or to assess target organ damage were not available in most facilities, particularly at the primary health-care level.<br>- Knowledge of blood pressure levels for hypertension was limited in about half of the non-physician health workers.<br>**Micro factors**- Almost 73% of patients had to pay fully out-of-pocket for consultation and/or drugs for the treatment of hypertension.<br>- About two-thirds of hypertensive patients utilized primary healthcare centres both for diagnosis and follow-up care.<br>- About half the patients were unaware of the beneficial effects of physical activity and non-smoking. Some were also unaware of the beneficial effects of a low-salt diet and a heart-healthy diet.
4. Capacity and site readiness for hypertension control program implementation in the Federal Capital Territory of Nigeria: a cross-sectional study<br>Ikechukwu A. Orji, 2021	60 primary healthcare facilities		To perform capacity and readiness assessments of public Primary Healthcare Centres (PHCs) to inform Nigeria's system-level hypertension control program's implementation and adaptation strategies	**Macro factors-** Areas with a higher density of training schools for community health extension workers had a relatively larger number of non-physician health workers in PHC centers.<br><br>**Meso factors-** most primary healthcare centres had sufficient human resource capacity, capacity for screening, diagnosis, and confirmation of hypertension.<br>- The availability of essential antihypertensive medicines was low.<br>- Few PHCs had guidelines, treatment algorithms, or information materials for hypertension diagnosis or management within the clinic on the assessment day.<br>- Few PHCs had received any training on diagnosing and

				managing cardiovascular diseases in the last 2 years.
5. Competition and quality of care under-regulated fees: evidence from Ghana<br>Adolf Kwadzo Dzampe, 2022	Administrative claims data of hypertension patients for 2017 – 2019 (36 months)		To examine how competition, measured by an increase in doctor-to-population ratio, affects the quality of care for hypertension patients in Ghana.<br><br>To investigate whether increased competition leads to higher or lower quality health outcomes, specifically focusing on in-hospital deaths and ACSC hospitalization rates.<br>To contribute new evidence and insights to the understanding of the relationship between competition and the quality of healthcare in the context of Ghana's healthcare system."	**Meso factors -** An increase in doctor density (measured by the doctor/population ratio) is associated with improved quality of hypertension care in Ghana.<br>- Increased doctor density leads to fewer hospitalizations for hypertensive patients and reduced in-hospital death rate, an indication of improved quality of care.<br>- Increased competition among physicians due to higher doctor density is suggested to incentivize health providers to improve the quality of care to attract more patients.
6. Determinants of high blood pressure and quality of Management in Three Regions of Benin<br>Mohamed Lamine Dramé, 2018.	27 PHC facilities (15 DHC, 5 CHC and 7 hospitals)<br>46 people interviewed<br>494 records or registry data.<br>51 patients<br><br><br>*** Private health centers		The objective of this work was to determine the prevalence of HBP in 2015 among adults aged 18 to 69 years living in the departments of Mono,<br>Couffo and Donga, to identify associated factors and to assess the quality of HBP<br>management in the PHC of these regions.	**Meso factors-** essential medicines such as calcium channel blockers and angiotensin-converting enzyme inhibitors for treating hypertension were unavailable in DHCs<br>- Basic materials for blood pressure measurement and for obesity screening were available in most of the PHCs.<br>- Care protocols and education materials for the prevention of cardiovascular diseases were scarcely available in these PHC, especially in the DHC.<br>- Most of the healthcare workers in the PHC have not received specific, recent professional training on hypertension.<br>**Micro factors-** All patients attending CHCs and almost all patients attending DHCs pay fully out-of-pocket for visits, care, and medications

	(PHCs), district health centers (DHC) and commune health centers (CHC)			
7. Drug utilization and blood pressure control in a population where antihypertensives are given free: effect of policy change.<br>ADA Adedapo, 2012.	116	adult hypertensive patients	To assess the current utilization pattern of antihypertensive drugs and blood pressure (BP) control among treated hypertensives where there is a change in payment policy for antihypertensive drugs and to compare with a previous study when drugs were given free in the same setting.	**Macro factors-** Fewer hypertensives were being followed up in this study compared with the previous study. This may be due to the drop in the catchment population and policy change. However, more patients achieved blood pressure control under the new payment policy.<br><br>The policy in 2009-2010 was changed to place a medical limit for each staff/ family member to a particular amount per annum which would be free, and the remaining cost of treatment would be paid by the patient. A form of co-payment. Also, an incentive of up to 50% of medical allowance was returned to any staff that did not fully use his/her allowance during the particular period.<br><br>**Micro factors-** Health education and patient counselling, were identified as crucial elements influencing the quality of care.
8. Enablers and barriers for implementing high-quality hypertension care in a rural primary care setting in Nigeria: perspectives of primary care staff and health insurance managers.<br>Aina O. Odusola, 2010	15 participants- 11 primary care staff and 4 health insurance staff	Primary care staff and health insurance managers	To explore the perspectives of primary care staff and health insurance managers on enablers and barriers to implementing high-quality hypertension care, in the context of a community-based health insurance program in rural Nigeria.<br>Specific objectives-<br>1) Access the current way in which hypertension care is provided; 2) Explore barriers that can make it difficult to	**Macro factors-** The KSHI (Kwara State Health Insurance) is an important facilitator for implementing high-quality hypertension care because it covers the cost of care for patients and provides essential resources and incentives to clinics: guidelines, staff training, medications, and diagnostic equipment. However, the cost of the services was not full covered by the insurance reimbursement system.<br><br>**Meso factors-** There is an absence of tools for tailored patient education at healthcare centre.<br>- Barriers to health insurance include late payment of claims, discordance between healthcare provider and insurer on how health insurance and provider payment

			provide high-quality hypertension care; and 3) Explore enablers that can facilitate the provision of (consistent) high-quality hypertension care.	methods work, and administrative challenges.<br>- Shortage of qualified health workers<br>**Micro factors-** Health insurance is affordable for most patients.
9. Essential medicines and technology for hypertension in primary healthcare facilities in Ebonyi State, Nigeria. Adeke AS, 2022	45 primary health facilities<br>145 patients	Hypertensive patients	- To assess the availability of essential medicines and technology in PHC facilities in Ebonyi State.<br>- To assess the affordability of essential medicines and technology in PHC facilities in Ebonyi State<br>- To measure the accessibility of essential medicines and technology in PHC facilities in Ebonyi State.	**Macro factors-** Factors such as long distances, bad road networks, and cost of transportation affect access to health facilities.<br>**Meso factors** – low availability of antihypertensive medications and complete lack in some of the facilities<br>- Some tools such as digital sphygmomanometers were unavailable in all facilities.<br>- Mercury sphygmomanometers, stethoscopes, weighing scales, and measuring tapes were available and functional in most facilities.<br>**Micro factors-** All the respondents could not afford their antihypertensive medications. The median monthly income was 8,000 Nigerian Naira (range = 2,000–52,000). The median monthly cost of antihypertensive medications was 3,500 Nigerian Naira (range = 1,500–10,000)
10. Social support and management of hypertension in South-west Nigeria. Osamor PE, 2015	440	hypertensive patients	1. To investigate the relationship between social support for treatment compliance among hypertensive subjects in a poor urban community in southwest Nigeria<br>2) To identify the correlates of social support in the study sample.	**Micro factors -** Individuals receiving support from friends were 55% more likely to adhere to treatment.<br>- The study found that friends' proactive involvement in hypertension awareness significantly impacted treatment compliance.<br>- Older age and marital status were linked to increased social support, influencing compliance.<br>- Marital status and religious affiliation emerged as key determinants influencing significant social support from family and friends.<br>- Having friends who were concerned about the respondent's hypertension or who helped remind the respondent about taking medication was associated with

				good compliance
11. The outpatient management of hypertension at two Sierra Leonean health centers: A mixed-method investigation of follow-up compliance and patient-reported barriers to care.<br>Herskind et al., 2020	68 adult patients of the hypertension treatment program<br><br>487 patient records	Hypertensive patients	This study sought to assess an initiative conducted by two health clinics to begin the treatment of hypertension amongst their patient populations by reviewing medication possession rates and documenting patient-reported barriers to care in the provision of chronic hypertension management.	**Macro factors-** The rainy season was mentioned as a barrier to access to health facilities as it affects the roads and makes transport to the clinics difficult.<br>**Micro factors-** A few patients mentioned home visits, outreaches, and call or mobile reminders as facilitators of quality patient care.<br>- Some patients cited that lowering the price for follow-up visits could improve patient attendance.<br>- Most patients cited transportation and financial difficulty as barriers to returning to the clinics. Other less-mentioned barriers included schedule conflicts with work or other prior commitments, forgetfulness, and lack of symptoms.<br>- About 36.8% of hypertensive patients did not attend follow-up appointments, 32.2% of patients attended one follow-up appointment and 30.9% of patients attended two or more follow-up appointments.
12. "We just have to help": Community health workers' informal task-shifting and task-sharing practices for hypertension and diabetes care in Nigeria.<br>Whenayon Simeon Ajisegiri, 2023	76 community health workers and other local and state government stakeholders	Community health workers	To investigate the practices of community health workers (CHWs) in the context of hypertension and diabetes care in PHC facilities in Nigeria. The study explores how CHWs implement national guidelines, their challenges, and the factors influencing their roles in providing care for non-communicable diseases (NCDs) such as hypertension and diabetes.	**Macro factors-** Socioeconomic factors like income disparities and lack of health insurance were identified as barriers to access to care.<br>**Meso factors-** Challenges in the availability of medication and equipment, lead to improvised methods for assessing blood pressure and glucose levels, impacting the quality of care.<br>- CHWs sometimes initiate treatments beyond the recommended scope, due to the need to address unmet needs of the community. Policy constraints and medication availability limit their roles.<br>- Inconsistent interpretation of guidelines, limited medication supplies, and inadequate training contribute to variations in care practices.<br>- Inadequate supplies of equipment, poor infrastructure, and inadequate supervision are the most frequent barriers to the delivery of hypertension and diabetes care.<br>- The study highlights weak referral linkages and non-compliance with referral guidelines, leading to inadequate

				communication between primary healthcare and higher-level facilities.
13. Health system challenges to hypertension and related non-communicable diseases prevention and treatment: perspectives from Ghanaian stakeholders<br>Laar A.K. et al., 2019	55 informants	Patients, health care professionals, licensed chemical sellers (LCS), national and sub-national policymakers	To understand patient-level and contextual challenges associated with managing hypertension and related NCDs in a Ghanaian district.	**Macro factors-** Low funding for NCDs due to the majority of the health system allocation going to maternal, child health, and infectious disease programs.<br>- There are no defined sources of funds from the Ghanaian government for awareness creation, screening, and prevention of hypertension according to a policymaker.<br>- Inadequate logistics and financing limits the implementation of national policies for NCDs<br>- Health services for NCDs are less available in poorer urban communities.<br>- Health policy limitations do not allow nurses to prescribe most medications including anti-hypertensive drugs.<br>**Meso factors-** Lack of screening facilities' equipment and staff<br>- Hypertension is included in the national health insurance scheme, however, the delayed reimbursement by the national health insurance authority affects medication availability.<br>- Lack of required numbers of qualified health personnel especially in poorer communities.<br>- Unavailability of essential drugs at lower-level service delivery points.<br>**Micro factors-** Poor adherence to medications<br>- Some patients did not realize the medications were lifelong.<br>- Out-of-pocket costs, costs of transportation, and cost of NHIS premiums are barriers to accessing treatment.<br>- The desire for curative treatment leads to the use of traditional medicines.
14. Hypertension in rural communities in Delta State, Nigeria: Prevalence, risk factors and barriers to health	134	Adults with and without hypertension	To assess the prevalence of hypertension and associated risk factors amongst adults in three villages in the Ibusa community in	**Macro factors-** Transport shortages to health facilities.<br>**Meso factors-** Low funding for hospital management, shortage of staff, and drug and equipment shortages in clinics are barriers to hypertension management.

care<br>Mary I. Ofili, 2015			Delta State, Nigeria.<br><br>To find out the prominent individual risk factors that contribute to the incidence of hypertension, and the key barriers to the health care of hypertensive patients.	
15. Assessment of hypertension service availability in some primary health centers in Nigeria: a mixed-methods study.<br>Oluseyi Adejumo, 2023	18 PHC workers and 305 PHC facilities.		This study aimed to assess hypertension service availability in some PHCs in Nigeria and seek recommendations that could facilitate improved services from PHC workers.	**Macro factors-** Inequitable distribution of health care workers in urban, semi-urban, and rural areas. The urban and semiurban PHCs had a significantly higher number of high and middle-cadre health workers than the rural PHCs.<br>**Meso factors-** Lack of essential antihypertensive medications in PHC centers<br>- Only 22.3% of health workers across the facilities have had training in hypertension.<br>- Low availability of essential antihypertensive medications, such that some PHC workers use their personal funds to purchase medications and equipment.<br>- National treatment guidelines were more available in rural facilities.<br>- Some PHC workers did not provide hypertension services because it was beyond their level of training.<br>- Shortage of skilled healthcare workers
16. Medication adherence and blood pressure control: A preliminary assessment of the role of health insurance in Nigeria and Ghana<br>Tijani Idris Ahmad Oseni, 2023	109	Hypertension patients	This study sought to assess the current impact of health insurance coverage on medication adherence and blood pressure control of patients being managed for hypertension in Ghana and Nigeria.	**Meso factors -** Health insurance coverage was higher among Ghanaian participants, compared to Nigeria<br>- Participants with health insurance coverage were 2.6 times more likely to have controlled blood pressure than those without insurance coverage<br>- Participants with health insurance had better medication adherence compared with non-enrollees.
