# Supplementary material for: Factors influencing access, quality and utilisation of primary healthcare for patients living with hypertension in West Africa: a scoping review
Source: BMJ Open. 2024 Dec 20;14(12):e088718. doi: 10.1136/bmjopen-2024-088718 (PMC11667391; doi:10.1136/bmjopen-2024-088718)

Appendices

Appendix 1- Key concepts and alternative terms.

Search concepts	Alternatives terms
1. Factors	Socio-cultural, socio-economic, social, societal, health systems, individual, patient, community, political
2. Influence	Hinder, enable, promote, threaten, impact, control, motivate, affect,
3.Primary care	Screening, medications, counselling, patient education, prescription, outpatient visits, lifestyle, access, utilization, quality of care
4. Hypertension	High blood pressure, raised blood pressure, increased blood pressure, hypertensive disease, hypertension diagnosis, hypertension treatment, hypertension control, hypertension cascade, NCD management
5. West Africa	Ghana, Niger, Burkina Faso, Benin, Cape Verde, Côte D'Ivoire, Gambia, Guinea, Guinea-Bissau, Liberia, Mali, Mauritania, Nigeria, Senegal, Sierra Leone, Togo

Appendix 2- search strategy for various databases

Search terms for google scholar	comments	Findings
(hinder OR enable OR promote OR influence OR affect) AND Hypertension AND "quality of care" AND "primary care" AND "West Africa" hypertension -pregnancy, -gestational	Must include – hypertension<br><br>Exclude pregnancy, gestational	954
(hinder OR enable OR promote OR influence OR affect) AND Hypertension AND "quality of care" AND "primary care" AND "West Africa" "hypertension" -pregnancy, -gestational	Must include – hypertension<br><br>Exclude pregnancy, gestational	
(Access OR utilization) AND "high blood pressure" AND "quality of care" AND "primary care" AND "West Africa" "hypertension" -pregnancy, -gestation,	Must include – hypertension<br><br>Exclude pregnancy, gestational	
(hinder OR enable OR promote OR influence OR affect) AND (access OR utilization) AND "High blood pressure" AND "quality of care" AND "primary care" AND "West Africa" hypertension -pregnancy, -gestational	Must include – hypertension<br><br>Exclude pregnancy, gestational	
(Socio-cultural OR socioeconomic OR individual OR community OR “health system” OR “health financing”) AND (Access OR utilization) AND "high blood pressure" AND "quality of care" AND "primary care" AND "West Africa" hypertension -pregnancy, -gestational	Must include – hypertension<br><br>Exclude pregnancy, gestational	
Search query for PubMed / Embase		
((((hinder OR enable OR promote OR influence OR affect[MeSH Terms]) AND ((Hypertension[MeSH Terms]) OR (((((((("high blood pressure") OR ("raised blood pressure")) OR ("increased blood pressure")) OR ("hypertensive disease")) OR ("hypertension diagnosis")) OR ("hypertension treatment")) OR ("hypertension control")) OR ("hypertension cascade")) OR ("NCD management")))) AND ((primary care[MeSH Terms]) OR (((((((((screening) OR (medication*)) OR (counselling)) OR ("patient education")) OR (prescription)) OR ("outpatient visit*")) OR (lifestyle)) OR (access)) OR (utilisation)) OR ("quality of care")))) AND ((West Africa[MeSH Terms]) OR (((((((((((((((Togo) OR ("Sierra Leone")) OR (Senegal)) OR (Nigeria)) OR (Mauritania)) OR (Mali)) OR (Liberia)) OR ("Guinea-Bissau")) OR (Guinea)) OR (Gambia)) OR ("Côte D'Ivoire")) OR ("Cape Verde")) OR (Benin)) OR ("Burkina Faso")) OR (Niger)) OR (Ghana)))) OR (((((hinder OR enable OR promote OR influence OR affect[MeSH Terms]) AND ((Hypertension[MeSH Terms]) OR (((((((("high blood pressure") OR ("raised blood pressure")) OR ("increased blood pressure")) OR ("hypertensive disease")) OR ("hypertension diagnosis")) OR ("hypertension treatment")) OR ("hypertension		2474

control")) OR ("hypertension cascade")) OR ("NCD management")))) AND ((primary care[MeSH Terms]) OR ((((((((((screening) OR (medication*)) OR (counselling)) OR ("patient education")) OR (prescription)) OR ("outpatient visit*")) OR (lifestyle)) OR (access)) OR (utilisation)) OR ("quality of care")))) AND (((quality of health care[MeSH Terms]) OR (health care quality[MeSH Terms])) OR (health care quality, access, and evaluation[MeSH Terms]))) AND ((West Africa[MeSH Terms]) OR ((((((((((((((((Togo) OR ("Sierra Leone")) OR (Senegal)) OR (Nigeria)) OR (Mauritania)) OR (Mali)) OR (Liberia)) OR ("Guinea-Bissau")) OR (Guinea)) OR (Gambia)) OR ("Côte D'Ivoire")) OR ("Cape Verde")) OR (Benin)) OR ("Burkina Faso")) OR (Niger)) OR (Ghana))))		
((((Socio-cultural OR socioeconomic OR individual OR community OR "health system" OR "health Insurance") AND ((Hypertension[MeSH Terms]) OR ((((((((("high blood pressure") OR ("raised blood pressure")) OR ("increased blood pressure")) OR ("hypertensive disease")) OR ("hypertension diagnosis")) OR ("hypertension treatment")) OR ("hypertension control")) OR ("hypertension cascade")) OR ("NCD management")))) AND ((primary care[MeSH Terms]) OR ((((((((((screening) OR (medication*)) OR (counselling)) OR ("patient education")) OR (prescription)) OR ("outpatient visit*")) OR (lifestyle)) OR (access)) OR (utilisation)) OR ("quality of care")))) AND ((West Africa[MeSH Terms]) OR ((((((((((((((((Togo) OR ("Sierra Leone")) OR (Senegal)) OR (Nigeria)) OR (Mauritania)) OR (Mali)) OR (Liberia)) OR ("Guinea-Bissau")) OR (Guinea)) OR (Gambia)) OR ("Côte D'Ivoire")) OR ("Cape Verde")) OR (Benin)) OR ("Burkina Faso")) OR (Niger)) OR (Ghana)))) OR (((((Socio-cultural OR socioeconomic OR individual OR community OR "health system" OR "health Insurance") AND ((Hypertension[MeSH Terms]) OR ((((((((("high blood pressure") OR ("raised blood pressure")) OR ("increased blood pressure")) OR ("hypertensive disease")) OR ("hypertension diagnosis")) OR ("hypertension treatment")) OR ("hypertension control")) OR ("hypertension cascade")) OR ("NCD management")))) AND ((primary care[MeSH Terms]) OR ((((((((((screening) OR (medication*)) OR (counselling)) OR ("patient education")) OR (prescription)) OR ("outpatient visit*")) OR (lifestyle)) OR (access)) OR (utilisation)) OR ("quality of care")))) AND (((quality of health care[MeSH Terms]) OR (health care quality[MeSH		

Terms])) OR (health care quality, access, and evaluation[MeSH Terms]))) AND ((West Africa[MeSH Terms]) OR (((((((((((((((Togo) OR ("Sierra Leone")) OR (Senegal)) OR (Nigeria)) OR (Mauritania)) OR (Mali)) OR (Liberia)) OR ("Guinea-Bissau")) OR (Guinea)) OR (Gambia)) OR ("Côte D'Ivoire")) OR ("Cape Verde")) OR (Benin)) OR ("Burkina Faso")) OR (Niger)) OR (Ghana))))		
Search query for Scopus		
hypertension AND (hinder OR enable OR promote OR influe nee OR affect) AND managem ent AND ("primary care" OR "p rimary healthcare") AND "West Africa" OR ghana OR niger 0R "Burkina Faso" OR benin 0R "Cape Verde" OR "Cote D/3zap os;lvoire" OR gambia OR guin ea OR "Guinea-Bissau" OR libe ria OR mali OR mauritania 0 R nigeria OR senegal OR "Sier ra Leone" OR toga AND (LIMIT-TO (AFFILCOUNTRY, "Nigeri a") OR LIMIT-TO (AFFILCOUN TRY, "Ghana") OR LIMIT-T0 (AFFILCOUNTRY, "Benin") OR LIMIT-TO (AFFILCOUNTR Y, "Gambia") OR LIMIT-TO(AFFILCOUNTRY, "Burkina Faso") OR LIMIT-TO (AFFILCOUNTRY, "Senegal") OR LIMIT-T0 (AFFILCOUNTRY, "Liberia") OR LIMIT-TO (AFFILCOUNTRY, "Sierra Leone") OR LIMIT-T0 (AFFILCOUNTRY, "Cote d8{a pos;lvoire") OR LIMIT-TO (AFFI LCOUNTRY, "Togo") OR LIMI T-TO (AFFILCOUNTRY, "Mali") OR LIMIT-TO (AFFILCOUNTR Y, "Niger") OR LIMIT-TO (AFFILCOUNTRY, "Cape Verde") 0 R LIMIT-TO (AFFILCOUNTRY, "Guinea") OR LIMIT-TO(AFFILCOUNTRY, "Guinea-Bissau") OR LIMIT-TO (AFFILCOUNTRY, "Mauritania")) AND (LIMIT-TO (PUBYEAR, 2023) OR LI MIT-TO (PUBYEAR, 2022) OR LIMIT-TO (PUBYEAR, 2021) 0 R LIMIT-TO (PUBYEAR, 2020) OR LIMIT-TO (PUBYEAR, 201 9) OR LIMIT-TO (PUBYEAR, 2018) OR LIMIT-TO (PUBYEA R, 2017) OR LIMIT-TO (PUBY EAR, 2016) OR LIMIT-TO (PU BYEAR, 2015) OR LIMIT-T0 (PUBYEAR, 2014) OR LIMIT-TO (PUBYEAR, 2013) OR LI MIT-TO (PUBYEAR, 2012) OR LIMIT-TO (PUBYEAR, 2011) 0 R LIMIT-TO (PUBYEAR, 2010) OR LIMIT-TO (PUBYEAR, 200 9) OR LIMIT-TO (PUBYEAR, 2008) OR LIMIT-TO (PUBYEA R, 2007) OR LIMIT-TO (PUBY EAR, 2006) OR LIMIT-TO (PU BYEAR, 2005) OR LIMIT-T 0 (PUBYEAR, 2004) OR LIMI T-TO (PUBYEAR, 2003) OR LI MIT-TO (PUBYEAR, 2002) OR LIMIT-TO (PUBYEAR, 2001) 0 R LIMIT-TO (PUBYEAR, 200 0))		2374
hypertension AND (hinder OR enable OR promote OR influenee OR affect) AND ("quality of care") AND ("primary care" OR "primary healthcare") AND "West Africa" OR ghana OR nig er OR "Burkina Faso" OR beni n OR "Cape Verde" OR "Cote D’lvoire" OR gambia OR g uinea OR "Guinea-Bissau" OR liberia OR mali OR mauritania OR nigeria OR senegal OR "Sierra Leone" OR toga AND (LIMIT-TO (		

AFFILCOUNTRY,"Nigeria") OR LIMIT-TO (AFFIL COUNTRY, "Ghana") OR LIMI T-TO (AFFILCOUNTRY, "Beni n") OR LIMIT-TO (AFFILCOUN TRY, "Gambia") OR LIMIT-T0 (AFFILCOUNTRY, "Burkina F aso") OR LIMIT-TO (AFFILCOU NTRY, "Senegal") OR LIMIT-T0 (AFFILCOUNTRY, "Liberia") OR LIMIT-TO (AFFILCOUNTRY, "Sierra Leone") OR LIMIT-T0 (AFFILCOUNTRY, "Cote dll{apos;lvoire") OR LIMIT-TO (AFFI LCOUNTRY, "Togo") OR LIMI T-TO (AFFILCOUNTRY, "Mali") OR LIMIT-TO (AFFILCOUNTR Y, "Niger") OR LIMIT-TO (AFFILCOUNTRY, "Cape Verde") 0 R LIMIT-TO (AFFILCOUNTRY, "Guinea") OR LIMIT-TO(AFFILCOUNTRY, "Guinea-Bissau") OR LIMIT-TO (AFFILCOUNTRY, "Mauritania")) AND (LIMIT-TO (PUBYEAR, 2023) OR LI MIT-TO (PUBYEAR, 2022) OR LIMIT-TO (PUBYEAR, 2021) 0 R LIMIT-TO (PUBYEAR, 2020) OR LIMIT-TO (PUBYEAR, 201 9) OR LIMIT-TO (PUBYEAR, 2018) OR LIMIT-TO (PUBYEA R, 2017) OR LIMIT-TO (PUBY EAR, 2016) OR LIMIT-TO (PU BYEAR, 2015) OR LIMIT-T0 (PUBYEAR, 2014) OR LIMIT-TO (PUBYEAR, 2013) OR LI MIT-TO (PUBYEAR, 2012) OR LIMIT-TO (PUBYEAR, 2011) 0 R LIMIT-TO (PUBYEAR, 2010) OR LIMIT-TO (PUBYEAR, 200 9) OR LIMIT-TO (PUBYEAR, 2008) OR LIMIT-TO (PUBYEA R, 2007) OR LIMIT-TO (PUBY EAR, 2006) OR LIMIT-TO (PU BYEAR, 2005) OR LIMIT-T0 (PUBYEAR, 2004) OR LIMI T-TO (PUBYEAR, 2003) OR LI MIT-TO (PUBYEAR, 2002) OR LIMIT-TO (PUBYEAR, 2001) 0 R LIMIT-TO (PUBYEAR, 200 0))		
hypertension AND (hinder OR enable OR promote OR influenee OR affect) AND (utilization OR access) AND ("primary care" OR "primary healthcare") AND "West Africa" OR ghana 0R niger OR "Burkina Faso" OR benin OR "Cape Verde" OR "C ate Dll{apos;lvoire" OR gambia OR guinea OR "Guinea-Bissa u" OR liberia OR mali OR ma uritania OR nigeria OR senega I OR "Sierra Leone" OR toga AND (LIMIT-TO(AFFILCOUNTR Y, "Nigeria") OR LIMIT-TO(A FFILCOUNTRY, "Ghana") OR LIMIT-TO (AFFILCOUNTRY, "B enin") OR LIMIT-TO (AFFILCO UNTRY, "Gambia") OR LIMIT-TO (AFFILCOUNTRY, "Burkina Faso") OR LIMIT-TO (AFFILCOUNTRY, "Senegal") OR LIMIT- TO (AFFILCOUNTRY, "Liberi a") OR LIMIT-TO (AFFILCOUNTRY, "Sierra Leone") OR LIMIT-TO (AFFILCOUNTRY, "Coted ll{apos;lvoire") OR LIMIT-TO (A FFILCOUNTRY, "Togo") OR LI MIT-TO (AFFILCOUNTRY, "Mal i") OR LIMIT-TO (AFFILCOUNT RY, "Niger") OR LIMIT-TO(AFFILCOUNTRY, "Cape Verde") OR LIMIT-TO (AFFILCOUNTR Y, "Guinea") OR LIMIT-TO(AFFILCOUNTRY, "Guinea-Bissa u") OR LIMIT-TO (AFFILCOUNTRY, "Mauritania")) AND (LI MIT-TO (PUBYEAR, 2023) OR LIMIT-TO (PUBYEAR, 2022) 0 R LIMIT-TO (PUBYEAR, 2021) OR LIMIT-TO (PUBYEAR, 202 0) OR LIMIT-TO (PUBYEAR, 2019) OR LIMIT-TO (PUBYEAR,2018) OR LIMIT-TO (PUBYEA R, 2017) OR LIMIT-TO (PUBY EAR,		

2016) OR LIMIT-TO (PU BYEAR, 2015) OR LIMIT-T0 (PUBYEAR, 2014) OR LIMIT-TO (PUBYEAR, 2013) OR LI MIT-TO (PUBYEAR, 2012) OR LIMIT-TO (PUBYEAR, 2011) 0 R LIMIT-TO (PUBYEAR, 2010) OR LIMIT-TO (PUBYEAR, 200 9) OR LIMIT-TO (PUBYEAR, 2008) OR LIMIT-TO (PUBYEA R, 2007) OR LIMIT-TO (PUBY EAR, 2006) OR LIMIT-TO (PU BYEAR, 2005) OR LIMIT-T0 (PUBYEAR, 2004) OR LIMI T-TO (PUBYEAR, 2003) OR LI MIT-TO (PUBYEAR, 2002) OR LIMIT-TO (PUBYEAR, 2001) 0 R LIMIT-TO (PUBYEAR, 2000))		
hypertension AND (socio-cultural OR socioeconomic OR individual OR community OR "heaIth system" OR "health financing") AND management AND ("primary care" OR "primary he althcare") AND "West Africa" 0 R ghana OR niger OR "Burkin a Faso" OR benin OR "Cape Verde" OR "Cote D’lvoire" OR gambia OR guinea OR "Guinea-Bissau" OR liberia OR mali OR mauritania OR nigeria OR senegal OR "Sierra Leone" OR toga AND (LIMIT-TO (AFFI LCOUNTRY, "Nigeria") OR LI MIT-TO (AFFILCOUNTRY, "Ghana") OR LIMIT-TO (AFFILCOUNTRY, "Benin") OR LIMIT-T0 (AFFILCOUNTRY, "Gambia") OR LIMIT-TO (AFFILCOUNTRY, "Burkina Faso") OR LIMI T-TO (AFFILCOUNTRY, "Senegal") OR LIMIT-TO (AFFILCOUN TRY, "Liberia") OR LIMIT-T0 (AFFILCOUNTRY, "Sierra Leo ne") OR LIMIT-TO(AFFILCOUNTRY, "Cote d’lvoire") 0 R LIMIT-TO(AFFILCOUNTRY, "Togo") OR LIMIT-TO (AFFILC OUNTRY, "Mali") OR LIMIT-T0 (AFFILCOUNTRY, "Niger") OR LIMIT-TO (AFFILCOUNTRY, "CapeVerde") OR LIMIT-T0 (AFFILCOUNTRY, "Guinea") OR LIMIT-TO (AFFILCOUNTRY, "Guinea-Bissau") OR LIMIT-TO (AFFILCOUNTRY, "Mauritania")) AND (LIMIT-TO(PUBYE AR, 2023) OR LIMIT-TO (PUB YEAR, 2022) OR LIMIT-TO (P UBYEAR, 2021) OR LIMIT-T0 (PUBYEAR, 2020) OR LIMI T-TO (PUBYEAR, 2019) OR LI MIT-TO (PUBYEAR, 2018) OR LIMIT-TO (PUBYEAR, 2017) 0 R LIMIT-TO (PUBYEAR, 2016) OR LIMIT-TO (PUBYEAR, 2015) OR LIMIT-TO (PUBYEAR, 2014) OR LIMIT-TO (PUBYEAR,2013) OR LIMIT-TO (PUBYEA R, 2012) OR LIMIT-TO (PUBY EAR, 2011) OR LIMIT-TO (PU BYEAR, 2010) OR LIMIT-T0 (PUBYEAR, 2009) OR LIMI T-TO (PUBYEAR, 2008) OR LI MIT-TO (PUBYEAR, 2007) OR LIMIT-TO (PUBYEAR, 2006) 0 R LIMIT-TO (PUBYEAR, 2005) OR LIMIT-TO (PUBYEAR, 200 4) OR LIMIT-TO (PUBYEAR, 2003) OR LIMIT-TO (PUBYEAR, 2002) OR LIMIT-TO (PUBY EAR, 2001) OR LIMIT-TO (PUBYEAR , 2000))		
hypertension AND (socio-cultural OR socioeconomic OR individual OR community OR "heaIth system" OR "health financing") AND ("qualityofcare") AN D ("primary care" OR "primary healthcare") AND "West Africa" OR ghana OR niger OR "Burkina Faso" OR benin OR "Cape Verde" OR "Cote D’lvoire" OR gambia OR guinea OR "Guinea-Bissau" OR liberia OR mali OR mauritania OR nigeria OR senegal OR "Sierra Leon e" OR toga AND (LIMIT-TO(A FFILCOUNTRY,		

"Nigeria") OR LIMIT-TO (AFFILCOUNTRY , "Ghana") OR LIMIT-TO (AFFILCOUNTRY, "Benin") OR LIMIT-T0 (AFFILCOUNTRY, "Gambi a") OR LIMIT-TO (AFFILCOUNTRY, "Burkina Faso") OR LIMI T-TO (AFFILCOUNTRY, "Senegal") OR LIMIT-TO (AFFILCOUN TRY, "Liberia") OR LIMIT-T0 (AFFILCOUNTRY, "Sierra Leo ne") OR LIMIT-TO(AFFILCOUNTRY, "Cote d'lvoire") 0R LIMIT-TO(AFFILCOUNTRY, "Togo") OR LIMIT-TO (AFFILC OUNTRY, "Mali") OR LIMIT-T0 (AFFILCOUNTRY, "Niger") OR LIMIT-TO (AFFILCOUNTRY, "Cape Verde") OR LIMIT-T0 (AFFILCOUNTRY, "Guinea") OR LIMIT-TO (AFFILCOUNTRY, "Guinea-Bissau") OR LIMIT-TO (AFFILCOUNTRY, "Mauritania")) AND (LIMIT-TO(PUBYE AR, 2023) OR LIMIT-TO (PUB YEAR, 2022) OR LIMIT-TO (P UBYEAR, 2021) OR LIMIT-T0 (PUBYEAR, 2020) OR LIMI T-TO (PUBYEAR, 2019) OR LI MIT-TO (PUBYEAR, 2018) OR LIMIT-TO (PUBYEAR, 2017) 0 R LIMIT-TO (PUBYEAR, 2016) OR LIMIT-TO (PUBYEAR, 2015) OR LIMIT-TO (PUBYEAR, 2014) OR LIMIT-TO (PUBYEAR,2013) OR LIMIT-TO (PUBYEAR, 2012) OR LIMIT-TO (PUBYEAR, 2011) OR LIMIT-TO (PU BYEAR, 2010) OR LIMIT-T0 (PUBYEAR, 2009) OR LIMIT-TO (PUBYEAR, 2008) OR LIMIT-TO (PUBYEAR, 2007) OR LIMIT-TO (PUBYEAR, 2006) 0 R LIMIT-TO (PUBYEAR, 2005) OR LIMIT-TO (PUBYEAR, 2004) OR LIMIT-TO (PUBYEAR, 2003) OR LIMIT-TO (PUBYEAR, 2002) OR LIMIT-TO (PUBYEAR, 2001) OR LIMIT-TO (PUBYEAR , 2000))		
hypertension AND (socio-cultural OR socioeconomic OR individual OR community OR "heaIth system" OR "health financing") AND (utilization OR access) AND ("primary care" OR "primary healthcare") AND "West Africa" OR ghana OR niger OR "Burkina Faso" OR benin OR "Cape Verde" OR "Cote D'lvoire" OR gambia OR guinea OR "Guinea-Bissau" OR liberia OR mali OR mauritania OR nigeria OR senegal OR "Sierra Leone" OR toga AND (LIMIT-T0 (AFFILCOUNTRY, "Nigeria") OR LIMIT-TO (AFFILCOUNTR Y, "Ghana") OR LIMIT-TO(AFFILCOUNTRY, "Benin") OR LI MIT-TO (AFFILCOUNTRY, "Gambia") OR LIMIT-TO (AFFILCOUNTRY, "Burkina Faso") OR LI MIT-TO (AFFILCOUNTRY, "Sen egal") OR LIMIT-TO (AFFILCO UNTRY, "Liberia") OR LIMIT-T0 (AFFILCOUNTRY, "Sierra Leo ne") OR LIMIT-TO(AFFILCOUNTRY, "Cote d'lvoire") 0R LIMIT-TO (AFFILCOUNTRY, "Togo") OR LIMIT-TO (AFFILC OUNTRY, "Mali") OR LIMIT-T0 (AFFILCOUNTRY, "Niger") OR LIMIT-TO (AFFILCOUNTRY, "CapeVerde") OR LIMIT T0 (AFFILCOUNTRY, "Guinea") OR LIMIT-TO (AFFILCOUNTRY, "Guinea-Bissau") OR LIMIT TO (AFFILCOUNTRY, "Maurita nia")) AND (LIMIT-TO (PUBYE AR, 2023) OR LIMIT-TO (PUB YEAR, 2022) OR LIMIT-TO (P UBYEAR, 2021) OR LIMIT-T0 (PUBYEAR, 2020) OR LIMI T-TO (PUBYEAR, 2019) OR LI MIT-TO (PUBYEAR, 2018) OR LIMIT-TO (PUBYEAR, 2017) 0 R LIMIT-TO (PUBYEAR, 2016) OR LIMIT-TO		

(PUBYEAR, 2015) OR LIMIT-TO (PUBYEAR, 2014) OR LIMIT-TO (PUBYEAR,<br>2013) OR LIMIT-TO (PUBYEAR, 2012) OR LIMIT-TO (PUBY EAR, 2011) OR LIMIT-TO (PUBYEAR, 2010) OR LIMIT-T0 (PUBYEAR, 2009) OR LIMI T-TO (PUBYEAR, 2008) OR LIMIT-TO (PUBYEAR, 2007) OR LIMIT-TO (PUBYEAR, 2006) 0 R LIMIT-TO (PUBYEAR, 2005) OR LIMIT-TO (PUBYEAR, 200 4) OR LIMIT-TO (PUBYEAR, 2003) OR LIMIT-TO (PUBYEAR, 2002) OR LIMIT-TO (PUBY EAR, 2001) OR LIMIT TO (PUBYEAR , 2000))		
Search query for Cairn Info		
1. "Soins primaire" ET "hypertension" ET "Afrique de l'Ouest"<br>2. "Accès" ET "Hypertension" ET "Afrique de l'Ouest"<br>3. "Accès" ET "Soins primaire" ET "hypertension"<br>4. Accès aux soins" ET "soins primaires" ET hypertension" ET "Afrique de l'ouest"<br>5. "Facteurs" ET "hypertension" ET " Afrique de l'Ouest"<br>6. "Facteurs" ET "soins primaires" ET "hypertension" ET " Afrique de l'Ouest"<br>7. "Influence" ET "hypertension" ET "Afrique de l'Ouest"<br>8. "Hypertension" ET "Afrique de l'Ouest"<br>9. "Utilisation" ET " Soins primaires" ET "hypertension" ET "Afrique de l'ouest"<br>10. "Qualité" ET " Soins primaires" ET "hypertension" ET "Afrique de l'ouest"	Lengthy combinations of key terms yielded zero results and as such, search terms were broken down into smaller combinations. After several varying combinations of key words, some papers were repeated in search results.	44

Appendix 3 -Flow chart for the inclusion and exclusion of papers

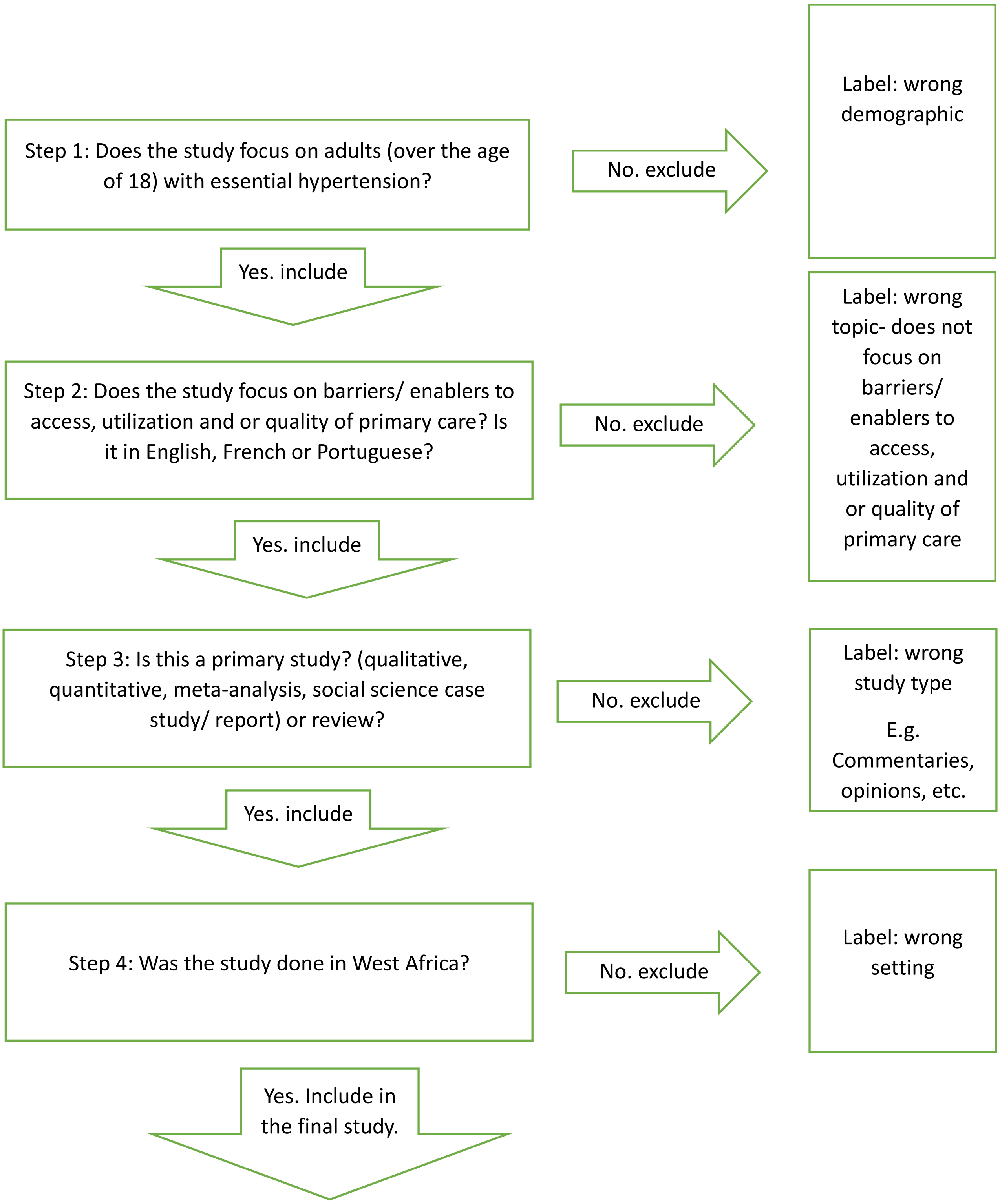

Figure 1- framework on the factors that influence primary care access, utilization, and quality for people living with hypertension

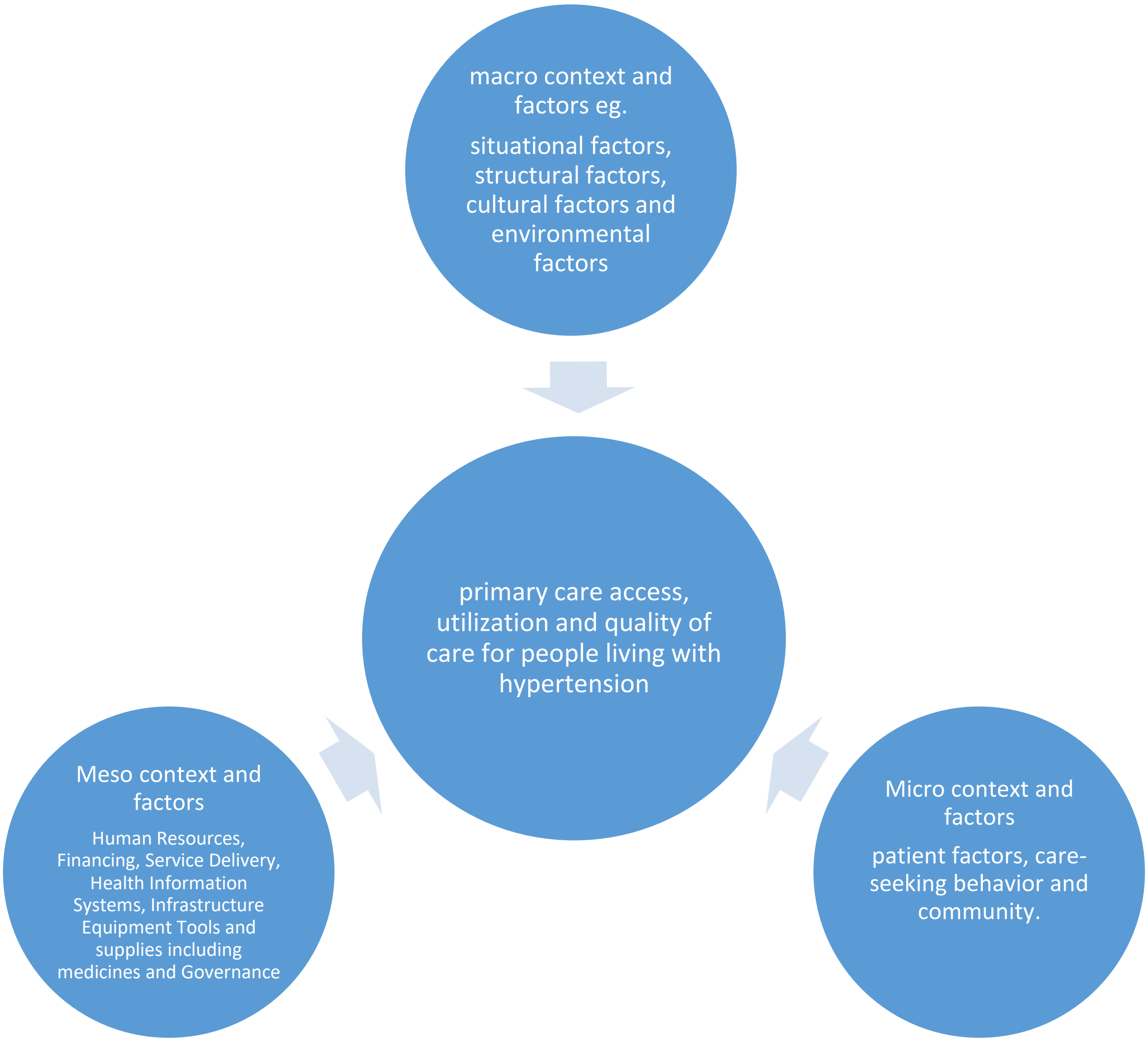

Figure 2- PRISMA 2020 FLOW diagram for scoping review

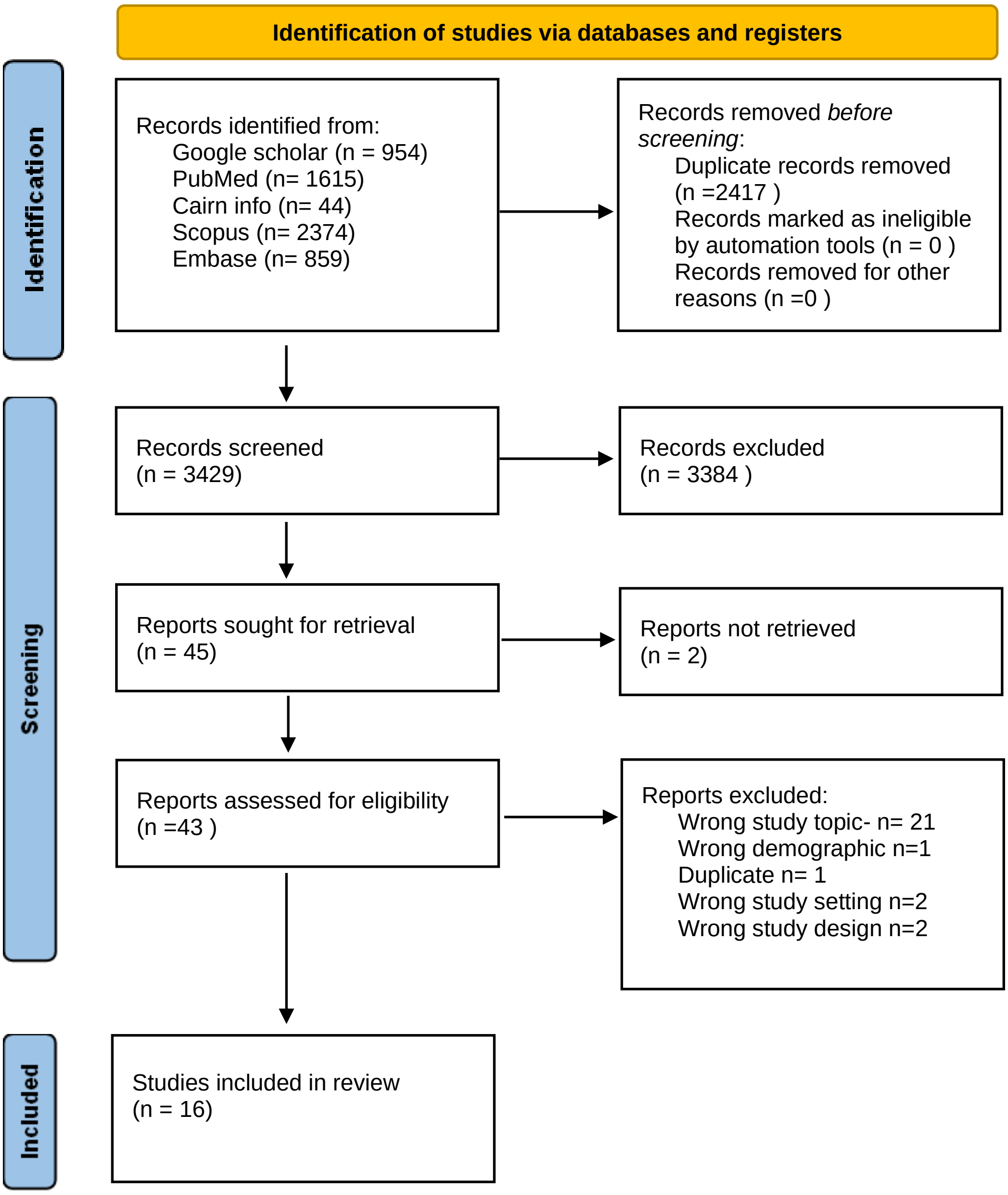

Figure 3- framework on the factors that influence primary care access, utilization, and quality for people living with hypertension showing interconnectedness of the factors.

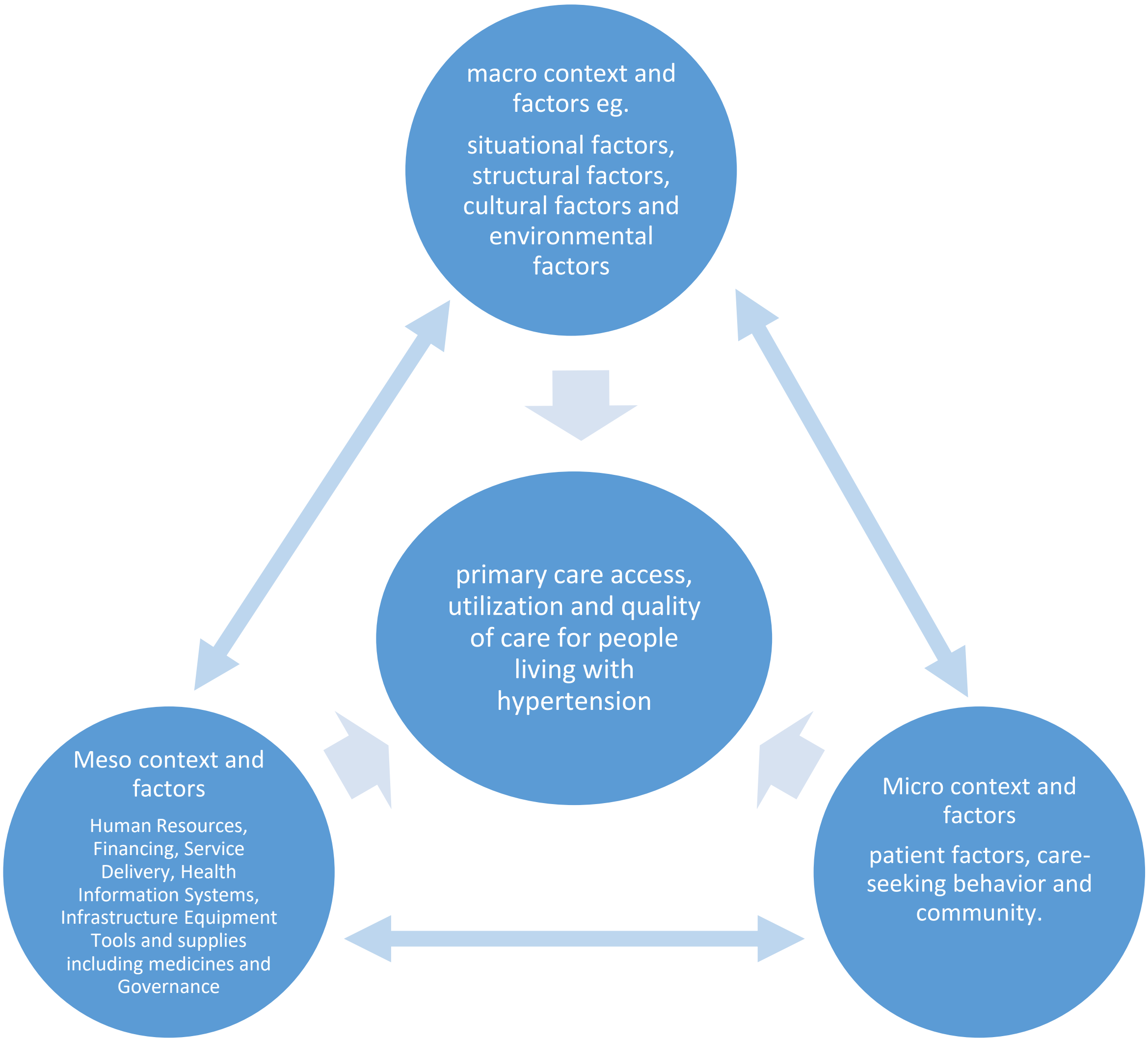

Supplement: online supplemental file 2 [file bmjopen-14-12-s002.pdf]
