# Supplementary material for: Factors influencing access, quality and utilisation of primary healthcare for patients living with hypertension in West Africa: a scoping review
Source: BMJ Open. 2024 Dec 20;14(12):e088718. doi: 10.1136/bmjopen-2024-088718 (PMC11667391; doi:10.1136/bmjopen-2024-088718)
Supplement: Supplementary file 3 [file bmjopen-2024-088718.reviewer_comments.pdf]

PEER REVIEW HISTORY

BMJ Open publishes all reviews undertaken for accepted manuscripts. Reviewers are asked to complete a checklist review form and are provided with free text boxes to elaborate on their assessment. These free text comments are reproduced below.

ARTICLE DETAILS

Title (Provisional)

Factors influencing access, quality, and utilization of primary health care for patients living with hypertension in West Africa - A scoping review.

Authors

Amarteyfio, Kezia Naa Amerley Akosua; Bondzie, Eugene Paa Kwesi; Reichenberger, Veronika; Afun, Nana Efua Enyimayew; Cofie, Albert Kofi Mensah; Agyekum, Mary Pomaa; Lamptey, Paul; Ansah, Evelyn K; Agyepong, Irene Akua; Mirzoev, Tolib; Perel, Pablo

---

VERSION 1 - REVIEW

Reviewer **1**

Name **Gerber, Felix**

Affiliation **University Hospital Basel, Department of Clinical Research, Division of Clinical Epidemiology**

Date **12-Aug-2024**

COI **None**

Dear authors,

Thank you very much for submitting this interesting manuscript.

It seems that a careful review of literature was done, and it is appreciated that the protocol was previously published. However, the writing of the manuscript seems to be done less carefully and I think major revisions are needed before publication. Please find detailed comments below.

General comments:

- Ensure consistent use of abbreviation, for example line 12: The abbreviation for Sub-Saharan Africa was introduced before, so it should be used instead of the full word

- Cite relevant, recent, scientific literature and try avoiding citing websites:

o Reference 3 : No author mentioned

o Reference 4 : No author and year mentioned

o Reference 6: No author mentioned

o References 8 and 9: Use more recent sources

- Correct punctuation. Sometimes you put a dot, then the brackets with references, then another dot, sometimes you omit one or the other dot. I would suggest ending a sentence, then put the brackets with the references and then the dot before starting a new sentence.

- Correct formatting. Different fonts are used for headers (for example “Introduction”, “Review Aims and Objectives”, and “Discussion” all have different fonts).

- The PRISMA-ScR checklist is not filled

- Strengths and Limitations should be included after the abstract not at the end of the manuscript

Abstract:

- The structure of the abstract should be adapted. It does not make sense to state “Studies were selected based on the inclusion and exclusion criteria” in the “Methods and analysis” section and then only mention those after the conclusion section. Please include all relevant information including eligibility criteria and language restrictions in the “Methods and analysis” section and end the abstract with the “Conclusion”

- The “Results” section should contain information on how many articles were identified, screened and included, and the most important characteristics of the included articles. The current content of the “Results” section rather belongs to a “Discussion” or “Conclusion” section. Also, it gives the impression that the authors already had a message in mind before doing the review and just want to transmit this.

- Explain which key barriers and enablers were identified based on your search.

Introduction:

- Page 5, Line 41: “Access and utilization of adequate quality primary healthcare for hypertension in West Africa is often quite low for multiple reasons such as cost, lack of knowledge, essential equipment, tools, and supplies as well as medicines.(17,18). Health system challenges such as insufficient tools, facilities, staff, and funding are documented…” The word tools is repeated in both sentences and also it is not clear what is meant by it as you mention essential equipment separately already.

- Page 5, Line 44: “Individual patient factors also affect how well their blood pressure is controlled”

o Specify which factors

Methods and Analysis

- Add the initials of authors doing the screening and identification of publications.

- Add reference for the PCCS framework

Results

- It is unclear whether 16 or 17 papers were included. In the flow chart it says 16, further down in the text 17. Please clarify.

- The results section is poorly structured. I am just giving examples here, but I think the whole “Results” and “Discussion” sections should be restructured.

o You mention the four dimensions of Leichter’s framework, but they are not addressed in a systematic way. For example, the cultural dimension is not mentioned at all

o Health insurance is mentioned in macro and meso level. Out-of-pocket expenditures are mentioned in meso and micro level. The framework is complex to understand for a reader, but it seems that not even the authors fully understand which factors to place in which category.

o Sentences like “Public Policies are decisions that become authoritative for society. Public policy decision-making is technical and political (process and power). What decisions are made as well as how they are implemented can influence primary care access, quality, and utilization” do not fit well in the “Results” section, where you should report the findings of the publications included in the review.

o You want to identify barriers and facilitators to primary level hypertension care. However, there is no structure in the text that enables the reader to quickly identify sections on specific facilitators or barriers.

o I would suggest rethinking the classification of different factors and then avoid duplication in several sections (for example the issue with health insurance). Furthermore, consider using sub-headings to facilitate overview and understanding (for example use sub-headings for the four dimensions of Leichter’s framework or for barriers and facilitators in each level). Also try to be more systematic in documenting how the publications included in the review support your statements (for example start a paragraph with “Four of the 16 studies addressed factors belonging to the situational dimension of Leichter’s framework (ref, ref, ref, ref). Two studies mentioned xxxx as relevant barriers (ref, ref). etc). You should ensure that the reader understands how many studies mentioned which barriers/facilitators in each level. This will also make it easier to understand the remaining gaps in the literature.

- Discussion and Conclusion:

o It is a valid and important finding, that factors belonging to the different levels are interconnected. However, you should explain better how you come to that conclusion based on the publications included in the review. Also, you should discuss your objectives in the “Discussion” section. You aimed at identifying factors at different levels that influence primary level hypertension care and analyze how and why they work. Currently, this is missing in the Discussion and Conclusion section.

o Discuss strengths and weaknesses of your approach in the “Discussion” section

o The Conclusion in the abstract does not have the same message as the Conclusion section in the main text.

**Reviewer**	**2**
**Name**	**Khoirunnisa, Sudewi Mukaromah**
**Affiliation**	**University of Groningen**
**Date**	**20-Aug-2024**
**COI**	**I have no conflicts of interest to declare**

---

1. General comments: Overall, the paper is a well-structured and comprehensive review that makes a significant contribution to understanding the challenges of hypertension care at the primary care level in West Africa. However, the manuscript is hard to follow, and some figures and tables were not cited correctly in the text.

2. Abstract:

a. Methods and Analysis: “This scoping review employs the approach described by Arksey and O’Malley in 2005”. This method did not mentioned somewhere in the main text. Please make it coherent between the abstract and the main text.

b. Methods and Analysis: A brief mention of the key inclusion and exclusion criteria used for study selection would enhance the transparency of the review process.

c. Results: The abstract should explicitly state the number of studies reviewed or the amount of data analyzed if possible.

3. Introduction:

a. The introduction provides a comprehensive overview of the topic, but the flow between the sections could be improved. Consider reordering some paragraphs to enhance readability. For example, the discussion on primary care might be more effective if introduced earlier, to establish the context before diving into statistics on hypertension.

b. The use of statistics is robust and helps to underline the severity of hypertension in Sub-Saharan Africa. However, some statistics are not fully contextualized. For instance, when mentioning the number of hypertensive individuals in Sub-Saharan Africa, it would be helpful to compare this to other regions or global figures to underscore the disparity.

c. The introduction does well to identify the gap in the literature—namely, the lack of studies focusing on West Africa. However, the justification for this study could be emphasized more. Why is it urgent to explore these barriers in West Africa specifically? Perhaps highlight the potential impact of this research on policy-making and public health interventions in the region.

d. The introduction of the conceptual framework is well done, but it could benefit from a brief explanation of why this specific framework (Leichter's framework) was chosen over others. Additionally, it would be useful to briefly describe how the framework will guide the analysis in the review.

4. The aims and objectives are clearly stated, but they could be more concise. Reducing redundancy, such as combining similar points, could make this section more straightforward. For instance, objectives 1.1, 1.2, and 1.3 could potentially be consolidated into a single objective that covers all contextual factors.

5. There are several technical terms used (e.g., "co-production of interventions"), which might need to be defined or explained for readers who are not specialists in the field. This will ensure the paper is accessible to a broader audience.

6. Method:

a. Mentioning Google Scholar, while useful, could be complemented by a justification of its inclusion alongside more traditional databases. This could help address potential concerns about the comprehensiveness and quality of the literature search.

b. Explain what is PCCS framework? Please explain the abbreviation

c. The use of the PCCS framework to guide the screening and identification of studies is appropriate. The framework is well-explained, but it could be strengthened by providing a rationale for choosing PCCS over other frameworks, such as PICO (Population, Intervention, Comparison, Outcome), which is more commonly used.

7. Results

a. P9 line 59. While the results include studies from several West African countries, there is a noticeable concentration on Nigeria (10 out of 16 studies). It might be useful to discuss the potential implications of this imbalance, such as whether the findings can be generalized across the region or if they are more reflective of the specific context in Nigeria.

b. P10 line 3. The issue of two papers not being available via open access is noted, but it would be beneficial to discuss how the absence of these studies might have impacted the overall results. Were these studies from underrepresented countries, or did they offer unique perspectives that might alter the conclusions?

c. P11 line 25. The analysis of public policy, especially the case from Nigeria, is insightful. However, the discussion on the policy change and its impact on utilization rates could be expanded. The potential influence of other variables, such as the drop in the catchment population, should be explored more deeply, perhaps through sensitivity analyses or additional data sources if available.

d. P12 line 11. The results highlight key barriers related to the location and distribution of health facilities, as well as transportation issues. This section would benefit from more detailed data, such as specific distances or travel times, to better quantify these barriers.

Additionally, discussing any innovative solutions or interventions that have been tried to overcome these barriers would add value.

e. P12 line 43. The section effectively highlights the correlation between provider knowledge, adherence to guidelines, and better care outcomes. However, it would be useful to mention whether there are any ongoing efforts to improve training and awareness among health workers, particularly in regions where knowledge gaps are prevalent. This could include national or regional training programs, continuing education initiatives, or efforts to standardize guidelines across different health facilities.

f. P12 line 50. The statistic that only 40% of non-physician health care providers were aware of the correct hypertension treatment goals is striking. Expanding on the implications of this knowledge gap for patient outcomes and exploring potential strategies to address this issue (e.g., regular training sessions or decision-support tools) would strengthen this section.

g. P12 line 58. The section discusses the variability in the availability of essential tools and supplies effectively. However, it would be beneficial to delve deeper into why these inconsistencies exist. Are they due to differences in regional funding, administrative efficiency, or other factors? Additionally, the mention of health workers using personal equipment is a significant point that could be expanded upon to emphasize the challenges faced by frontline workers and the potential risks to quality of care.

h. P13 line 21. The contrast between service readiness assessments and the actual reports from health workers in Nigeria is interesting. It might be useful to explore why these discrepancies exist and whether they suggest a need for more frequent or nuanced assessments that capture the reality of service provision on the ground.

i. P13 line 28. The positive impact of health insurance on hypertension control is well-noted. However, the section could be strengthened by discussing the broader implications of low health insurance coverage. For instance, what are the systemic barriers preventing wider coverage? Is there a lack of awareness, affordability issues, or administrative hurdles? Exploring these questions could provide a more rounded perspective on the role of health insurance in hypertension management.

j. While the results are presented clearly, the section could benefit from a more comparative analysis across the different countries studied. For example, how do the findings from Nigeria compare to those from Ghana or Benin in terms of human resources, equipment availability, or health financing? Such a comparison would provide a more holistic view of the challenges and potential solutions across West Africa.

k. The section would be strengthened by explicitly discussing the policy implications of these findings. How can the insights gained from this analysis inform future health system strengthening efforts in West Africa? Are there specific recommendations that emerge from these findings that could guide policymakers, healthcare administrators, or international organizations in addressing the identified barriers?

l. P14 line 25. While the section provides a good overview of the factors, the analysis could be deepened. For instance, the impact of financial barriers on long-term health outcomes could be explored further, possibly by linking it to broader economic factors or healthcare policies. Additionally, more discussion on how patient education programs can be designed to overcome the identified knowledge gaps would be valuable.

VERSION 1 - AUTHOR RESPONSE

Reviewer 1	
General comments:<br>- Ensure consistent use of abbreviation, for example, line 12: The abbreviation for Sub-Saharan Africa was introduced before, so it should be used instead of the full word<br>- Cite relevant, recent, scientific literature and try avoiding citing websites:<br>o Reference 3 : No author mentioned<br>o Reference 4 : No author and year mentioned<br>o Reference 6: No author mentioned<br>o References 8 and 9: Use more recent sources	We thank the reviewer for the suggestions, and we have now corrected the use of abbreviations throughout the manuscript to ensure consistency<br><br>References 3, 4 and 6 have been corrected.<br>We have included a more recent source for reference 8, however we did not find a recent version of reference 9.<br>Page 5 line 8
- Correct punctuation. Sometimes you put a dot, then the brackets with references, then another dot, sometimes you omit one or the other dot. I would suggest ending a sentence, then put the brackets with the references and then the dot before starting a new sentence.	Thank you, the punctuation has been corrected throughout the document. We have edited the position of the reference to before the dot as suggested.
- Correct formatting. Different fonts are used for headers (for example “Introduction”, “Review Aims and Objectives”, and “Discussion” all have different fonts).	Consistent fonts have now been used for all headings
- The PRISMA-ScR checklist is not filled	The checklist has now been filled
- Strengths and Limitations should be included after the abstract not at the end of the manuscript	Strengths and Limitations have now been included immediately after the abstract. Page 4
Abstract:<br>- The structure of the abstract should be adapted. It does not make sense to state “Studies were selected based on the inclusion and exclusion criteria” in the “Methods and analysis” section and then only mention those after the conclusion section. Please include all relevant information including eligibility criteria and language restrictions in the “Methods and Analysis” section and end the abstract with the “Conclusion”	Thank you, we have now adapted the abstract structure as suggested by the reviewer.<br>Page 3
- The “Results” section should contain information on how many articles were identified, screened and	We thank the reviewer for this useful recommendation, and we

included, and the most important characteristics of the included articles. The current content of the "Results" section rather belongs to a "Discussion" or "Conclusion" section. Also, it gives the impression that the authors already had a message in mind before doing the review and just want to transmit this.	have now revised the results and discussion sections accordingly.
- Explain which key barriers and enablers were identified based on your search.	Thank you, the key barriers and enablers have been indicated in the results section as suggested.
Introduction:<br>- Page 5, Line 41: "Access and utilization of adequate quality primary healthcare for hypertension in West Africa is often quite low for multiple reasons such as cost, lack of<br>knowledge, essential equipment, tools, and supplies as well as medicines. (17,18). Health system challenges such as insufficient tools, facilities, staff, and funding are documented..." The word tools is repeated in both sentences and also it is not clear what is meant by it as you mention essential equipment separately already.	Thank you, this has been corrected and the text has been revised to avoid repetition. Please find on Page 5 line 20
- Page 5, Line 44: "Individual patient factors also affect how well their blood pressure is controlled"<br>o Specify which factors	Thank you, the factors have now been listed. Please find on page 5 line 21)
Methods and Analysis<br>- Add the initials of authors doing the screening and identification of publications.	Thank you, the initials of the authors who did the screening and identification of publications have been included in the methods. Please find on page 8 line 5 and page 7 line 2
- Add reference for the PCCS framework	We have now included the reference for the PCCS as suggested. (please see page 8 line 13)
Results<br>a. It is unclear whether 16 or 17 papers were included. In the flow chart it says 16, further down in the text 17. Please clarify.	We apologize for the typo. The correct number is 16 included papers. We have now edited the text accordingly.
b. The results section is poorly structured. I am just giving examples here, but I think the whole "Results" and "Discussion" sections should be restructured.<br>o You mention the four dimensions of Leichter's framework, but they are not addressed in a systematic way. For example, the cultural dimension is not mentioned at all	b. Thank you for the comment. We have restructured the results and discussion section as suggested. Some sections of the Leichter's framework were left out of the results because there were no findings in those categories from the studies included. We have included a sentence to indicate this in the results section. Please see

	Page 21 line 7
c. Health insurance is mentioned in macro and meso level. Out-of-pocket expenditures are mentioned in meso and micro level. The framework is complex to understand for a reader, but it seems that not even the authors fully understand which factors to place in which category.	c. Factors related to health insurance were present in Macro and Meso levels because the various components of it are influenced differently at each level.
d. Sentences like “Public Policies are decisions that become authoritative for society. Public policy decision-making is technical and political (process and power). What decisions are made as well as how they are implemented can influence primary care access, quality, and utilization” do not fit well in the “Results” section, where you should report the findings of the publications included in the review.	d. We thank the reviewer for the suggestion and we have now removed this specific sentence from the result section.
e. You want to identify barriers and facilitators to primary level hypertension care. However, there is no structure in the text that enables the reader to quickly identify sections on specific facilitators or barriers.	Thank you for the suggestion. On balance, we felt that including sub-headings on facilitators and barriers under each of the six building blocks would unnecessarily fragment the Results section so instead we have now identified facilitators and barriers more explicitly within the current headings.
f. I would suggest rethinking the classification of different factors and then avoiding duplication in several sections (for example the issue with health insurance). Furthermore, consider using sub-headings to facilitate overview and understanding (for example use sub-headings for the four dimensions of Leichter’s framework or for barriers and facilitators in each level).	We thank the reviewer for this useful suggestion while we retained the three-tier structure of the context (macro, meso, micro) which we think gives a good structure to the result section, we have now highlighted relevant factors in italics which we hope provides greater clarity. As not all dimensions of the Leichter’s framework were included in the studies of our review, we believe it is better not to follow that structure as some of the headings would be “empty”
Also try to be more systematic in documenting how the publications included in the review support your statements (for example start a paragraph with “Four of the 16 studies addressed factors belonging to the situational dimension of Leichter’s framework (ref, ref, ref, ref). Two studies mentioned xxxx as relevant barriers (ref, ref). etc). You should ensure that the reader understands how many studies mentioned which	Thank you and as recommended, the specific number of studies mentioning specific barriers or facilitators have been included in the results. For example in<br>Page 21 line 11 and line 29<br>Page 22 line 15 and line 1

barriers/facilitators in each level. This will also make it easier to understand the remaining gaps in the literature.	
- Discussion and Conclusion:<br>o It is a valid and important finding, that factors belonging to the different levels are interconnected. However, you should explain better how you come to that conclusion based on the publications included in the review. Also, you should discuss your objectives in the "Discussion" section. You aimed at identifying factors at different levels that influence primary level hypertension care and analyze how and why they work. Currently, this is missing in the Discussion and Conclusion section.	Thank you for highlighting this, and we have now included more explicit results highlighting the interconnectedness (see page 22 line 23) and have also elaborated on this in the Discussion and Conclusions.
o Discuss strengths and weaknesses of your approach in the "Discussion" section	We agree that is an important aspect of our review and we have now included a section detailing the strengths and weaknesses of the study (second last paragraph of the Discussion. Page 26 line 2)
o The Conclusion in the abstract does not have the same message as the Conclusion section in the main text.	Thank you and the conclusions have now been aligned.
Reviewer 2	
Abstract:<br>a. Methods and Analysis: "This scoping review employs the approach described by Arksey and O'Malley in 2005". This method did not mentioned somewhere in the main text. Please make it coherent between the abstract and the main text.	a. Thank you for the opportunity to clarify this methodological aspect of our study. The approach by Askey and O'Malley was detailed in the published protocol. A link and reference to the protocol paper have been provided in the methods and analysis section, and in addressing this comment we have just removed reference in the Abstract.
b. Methods and Analysis: A brief mention of the key inclusion and exclusion criteria used for study selection would enhance the transparency of the review process.	b. Thank you, the key inclusion and exclusion criteria used for study selection has been included in the eligibility criteria of the abstract.
c. Results: The abstract should explicitly state the number of studies reviewed or the amount of data analyzed if possible.	c. We agree with the suggestion and we have now added the number of studies reviewed and the amount of data analyzed in the results section.
Introduction:<br>a. The introduction provides a comprehensive overview of the topic, but the flow between the sections could be improved. Consider reordering some paragraphs to	Thank you for your positive feedback, as suggested we have now reordered the paragraphs to enhance readability.

enhance readability. For example, the discussion on primary care might be more effective if introduced earlier, to establish the context before diving into statistics on hypertension.	
b. The use of statistics is robust and helps to underline the severity of hypertension in Sub-Saharan Africa. However, some statistics are not fully contextualized. For instance, when mentioning the number of hypertensive individuals in Sub-Saharan Africa, it would be helpful to compare this to other regions or global figures to underscore the disparity.	b. Thank you, following your suggestion, global statistics have now been included for comparison, please see page 5 line 4
c. The introduction does well to identify the gap in the literature—namely, the lack of studies focusing on West Africa. However, the justification for this study could be emphasized more. Why is it urgent to explore these barriers in West Africa specifically? Perhaps highlight the potential impact of this research on policy-making and public health interventions in the region.	c. Thanks for the excellent suggestion to make a stronger case for our study. We have now articulated the rationale for this paper more clearly in the last paragraph of the Introduction
d. The introduction of the conceptual framework is well done, but it could benefit from a brief explanation of why this specific framework (Leichter's framework) was chosen over others. Additionally, it would be useful to briefly describe how the framework will guide the analysis in the review.	d. Thank you, following the reviewer's suggestion we have now described the framework in more detail and explained why it was selected in the paragraph before the Methods
The aims and objectives are clearly stated, but they could be more concise. Reducing redundancy, such as combining similar points, could make this section more straightforward. For instance, objectives 1.1, 1.2, and 1.3 could potentially be consolidated into a single objective that covers all contextual factors.	Thanks for the useful suggestion to make our study clearer. We have now simplified the objectives and consolidated the objectives 1.1-1.3 into a single objective as suggested.<br>Page 6 line 7
There are several technical terms used (e.g., "co-production of interventions"), which might need to be defined or explained for readers who are not specialists in the field. This will ensure the paper is accessible to a broader audience	Thank you for raising this potential source of confusion for the reader. To enhance the readability, we have now removed the term 'co-production'
Method:<br>a. Mentioning Google Scholar, while useful, could be complemented by a justification of its inclusion alongside more traditional databases. This could help address potential concerns about the comprehensiveness and quality of the literature search.	Thank you, and we have explained in the Methods that the inclusion of Google Scholar was driven by the rationale to capture some of the newer journals which may not have been captured in traditional databases.<br>Page 7 line 21
b. Explain what is PCCS framework? Please explain the abbreviation	b. Thank you, the abbreviation has been explained and a reference provided<br>page 8 line 12

	the PCCS framework stands for Population, Concept, Context, and Study elements.
c. The use of the PCCS framework to guide the screening and identification of studies is appropriate. The framework is well-explained, but it could be strengthened by providing a rationale for choosing PCCS over other frameworks, such as PICO (Population, Intervention, Comparison, Outcome), which is more commonly used.	c. As recommended, the rationale for choosing the PCCS framework over other frameworks has been explained on page 8 line 13
Results<br>a. P9 line 59. While the results include studies from several West African countries, there is a noticeable concentration on Nigeria (10 out of 16 studies). It might be useful to discuss the potential implications of this imbalance, such as whether the findings can be generalized across the region or if they are more reflective of the specific context in Nigeria.	a. Thank you for raising this important finding of our review. The potential implications of the imbalance of countries and its impact on generalizability across the regions have been discussed on page 23 line 26<br>Most studies found were from Ghana and Nigeria, which limits the generalizability of the review findings.
b. P10 line 3. The issue of two papers not being available via open access is noted, but it would be beneficial to discuss how the absence of these studies might have impacted the overall results. Were these studies from underrepresented countries, or did they offer unique perspectives that might alter the conclusions?	b. Thank you, the studies were from Nigeria and Senegal. We have now highlighted the possible impact these studies could have made if included in the discussion section on page 26 line 9<br>The findings from the study in Nigeria would have shed more light on the patient and caretakers' experiences with hypertension and the study from Senegal would shed more light on an underrepresented country. However, it is likely the findings would have been similar and complemented those from the included studies.
c. P11 line 25. The analysis of public policy, especially the case from Nigeria, is insightful. However, the discussion on the policy change and its impact on utilization rates could be expanded. The potential influence of other variables, such as the drop in the catchment population, should be explored more deeply, perhaps through sensitivity analyses or additional data sources if available.	Thank you, we have now expanded the discussion as recommended. Unfortunately, the original paper did not have enough data to conduct a sensitivity analysis.

d. P12 line 11. The results highlight key barriers related to the location and distribution of health facilities, as well as transportation issues. This section would benefit from more detailed data, such as specific distances or travel times, to better quantify these barriers. Additionally, discussing any innovative solutions or interventions that have been tried to overcome these barriers would add value.	d. Thank you, more detailed data about the travel times have been included. (Page 21 line 3) However, exploring interventions to overcome the barriers is outside the scope of our study.
e. P12 line 43. The section effectively highlights the correlation between provider knowledge, adherence to guidelines, and better care outcomes. However, it would be useful to mention whether there are any ongoing efforts to improve training and awareness among health workers, particularly in regions where knowledge gaps are prevalent. This could include national or regional training programs, continuing education initiatives, or efforts to standardize guidelines across different health facilities.	Thank you. The study which was referenced in this point did not highlight any interventions to improve knowledge gaps among health care providers.
f. P12 line 50. The statistic that only 40% of non-physician health care providers were aware of the correct hypertension treatment goals is striking. Expanding on the implications of this knowledge gap for patient outcomes and exploring potential strategies to address this issue (e.g., regular training sessions or decision-support tools) would strengthen this section.	f. Thank you, potential strategies to address the issue of health care provider knowledge gap has been included on page 25 line 4
g. P12 line 58. The section discusses the variability in the availability of essential tools and supplies effectively. However, it would be beneficial to delve deeper into why these inconsistencies exist. Are they due to differences in regional funding, administrative efficiency, or other factors? Additionally, the mention of health workers using personal equipment is a significant point that could be expanded upon to emphasize the challenges faced by frontline workers and the potential risks to quality of care.	We take this point, and while there is insufficient data reported in the reviewed studies to delve deeper into each context, we have reflected more on the potential inter-relationships between different factors.<br>Page 24 line 14
h. P13 line 21. The contrast between service readiness assessments and the actual reports from health workers in Nigeria is interesting. It might be useful to explore why these discrepancies exist and whether they suggest a need for more frequent or nuanced assessments that capture the reality of service provision on the ground.	Thank you, as mentioned above, we summarized findings from the included studies. Although the reasons for these discrepancies were not reported in the included paper, we have included possible reasons for the discrepancies in service readiness assessments and the actual reports from health workers on page 24 line 30
i. P13 line 28. The positive impact of health insurance on hypertension control is well-noted. However, the section could be strengthened by discussing the broader implications of low health insurance coverage. For instance, what are the systemic barriers preventing wider	i. we take this point, and the possible reasons for low health insurance coverage have been included on page 24 line 20

coverage? Is there a lack of awareness, affordability issues, or administrative hurdles? Exploring these questions could provide a more rounded perspective on the role of health insurance in hypertension management.	
j. While the results are presented clearly, the section could benefit from a more comparative analysis across the different countries studied. For example, how do the findings from Nigeria compare to those from Ghana or Benin in terms of human resources, equipment availability, or health financing? Such a comparison would provide a more holistic view of the challenges and potential solutions across West Africa.	j. Thank you, based on the reported studies the challenges appear to be similar in all countries. We did not include this in the results section to maintain the current structure and enhance readability. However, in addressing this comment, a section on this has been included in the discussion on page 24 line 14
k. The section would be strengthened by explicitly discussing the policy implications of these findings. How can the insights gained from this analysis inform future health system-strengthening efforts in West Africa? Are there specific recommendations that emerge from these findings that could guide policymakers, healthcare administrators, or international organizations in addressing the identified barriers?	k. Thank you for your suggestion, one key policy implication of the study findings was discussed in the last paragraph of the discussion section.<br>Page 18 line 23
l. P14 line 25. While the section provides a good overview of the factors, the analysis could be deepened. For instance, the impact of financial barriers on long-term health outcomes could be explored further, possibly by linking it to broader economic factors or healthcare policies. Additionally, more discussion on how patient education programs can be designed to overcome the identified knowledge gaps would be valuable.	We thank the reviewer for this useful suggestion that we have followed and in doing so we think we have strengthen the discussion section. We have now deepened these aspects in the Discussion (e.g. the fourth paragraph of Discussion) section.
